# Dynamic progression of ectopic lymphoid structure formation in lacrimal glands of a Sjögren’s disease murine model

**DOI:** 10.3389/fimmu.2026.1797691

**Published:** 2026-03-19

**Authors:** Sara Abdelhamid, Alison V. Ramirez, Emre Aksan, Elizaveta A. Demianova, Cintia S. de Paiva, Maria C. Edman, J. Andrew MacKay, Sarah F. Hamm-Alvarez

**Affiliations:** 1 Department of Pharmacology and Pharmaceutical Sciences, Mann School of Pharmacy and Pharmaceutical Sciences, University of Southern California, Los Angeles, CA, United States; 2 Department of Ophthalmology, Keck School of Medicine, University of Southern California, Los Angeles, CA, United States; 3 Ocular Surface Center, Department of Ophthalmology, Baylor College of Medicine, Houston, TX, United States; 4 Department of Biomedical Engineering, Viterbi School of Engineering, University of Southern California, Los Angeles, CA, United States

**Keywords:** apoptosis, autoimmune disease, ferroptosis, lacrimal gland, secondary lymphoid organs, Sjögrens disease, ectopic lymphoid structures

## Abstract

**Background:**

Sjögren’s Disease (SjD) is a chronic autoimmune condition characterized by lymphocytic infiltration of lacrimal glands (LG) and salivary glands (SG). In SG, these immune structures have properties of ectopic lymphoid structures (ELS) and appear to play a critical role in disease pathology. While the presence of ELS in patients’ SG biopsies is linked to disease severity, their presence and composition in LG has not been well characterized.

**Methods:**

The properties and time course of apparent ELS development in LG from the male non-obese diabetes free (NOR) sub-strain of the Non-Obese Diabetic (NOD) mice was investigated at stages encompassing early to advanced lymphocytic infiltration. LG ELS were characterized morphologically by histology and immunofluorescence. Changes in LG gene expression were determined for genes involved in ELS formation and maturation, LG homeostasis and LG apoptosis.

**Results:**

LG ELS were characterized by segregation of B and T cell zones and the presence of high endothelial venules, follicular dendritic centers, GL7+ germinal center B cells, and IgG producing plasma cells. Gene expression data showed early upregulation of ELS indicators such as *Ltb, Glycam1, Cxcl13, Cxcr5, Ccl19 and Ccr7* while other components showed elevation at later stages (*Aicda* and *Il21*). Increased *Fasl* and decreased *Gpx4* expression suggested glandular apoptosis and ferroptosis concomitant with ELS formation.

**Conclusion:**

ELS form spontaneously in the LG of the male NOR mouse model of SjD and can recapitulate features of secondary lymphoid organs potentially acting as drivers of autoimmunity to sustain local glandular disease.

## Introduction

Ectopic lymphoid structures (ELS) are dynamic immune assemblies that develop transiently and postnatally in non-lymphoid tissues or organs during localized infection or in areas surrounding solid tumors. In a healthy immune environment, these structures exert protective effects to eliminate pathogens or eradicate cancer, respectively, and are resolved once antigens are eliminated (1). In the context of autoimmune diseases, ELS can persist as a defining characteristic of the affected tissue and contribute to pathogenesis.

The autoimmune disease, Sjögren’s Disease (SjD), is characterized by lymphocytic infiltration of the lacrimal (LG) and salivary (SG) glands, associated with development of severe dry eye and dry mouth, respectively (2). Diverse extra-glandular manifestations including inflammatory arthritis, interstitial lung disease, cutaneous vasculitis, pancreatic dysfunction, and peripheral neuropathy are also seen in many SjD patients (3). The presence of ELS in SG biopsies of SjD patients is correlated with high levels of circulating autoantibodies, systemic disease manifestations and most importantly, development of B-cell lymphoma (4). B-cell lymphoma occurs in 5% of SjD patients (1, 5), and identification of ELS from SG biopsies by histology is a prognostic tool for prediction of lymphoma development in SjD patients (5). LG biopsy tissue is not readily available from patients, and much less is known about ELS formation in the LG in SjD in either human disease or murine models. Although SjD affects both LG and SG, their properties including the organization of acinar and ductal epithelial cells, the nature of their secretions, and their mechanisms of tissue regeneration through stem cells are distinct (6). Disease pathology including ELS formation in each type of gland may thus vary.

The focus of this study was to characterize the formation, progression and composition of immune infiltrates in the LG associated with SjD-like autoimmune dacryoadenitis and to determine whether these infiltrates resemble ELS. Astorri et al. (7) and others have shown that ELS acquire functional and morphological features of secondary lymphoid organs (SLO). Here we have analyzed these features in the LG including the development of: 1) high endothelial venules (HEV) that recruit naïve B and T cells; 2) B and T cell aggregates with clear segregation; and 3) follicular dendritic cell networks (FDC) associated with auto-activation, clonal expansion of B cells and their differentiation into high-affinity, autoantibody-producing plasma cells (7, 8).

The non-obese diabetic resistant NOR/LtJ (NOR) mouse, a substrain of the non-obese diabetic (NOD) mouse, was used for these studies. NOR mice arose from genetic contamination of inbred NOD/ShiLtJ mice with C57BL/KsJ at the Jackson laboratory (9). NOD mice are a well-established SjD model that share many characteristics of human SjD (10–13). However, NOD mice develop autoimmune exocrinopathy at a young age which overlaps with their development of type 1 diabetes (10, 14, 15). This rapid disease onset and confounding effects of diabetes complicates the detailed evaluation of SjD pathology in the LG and SG of NOD mice. In contrast, NOR mice exhibit a slower SjD progression, making this model more representative of human autoimmune disease (10). Like the NOD mice, NOR mice display sex differences in SjD disease development, with males developing primarily LG disease and females developing primarily SG disease in the submandibular gland (SMG) (16, 17). With our focus on ELS formation in the LG, the male NOR mouse was primarily used for these studies.

In male NOR mice, LG ELS form as early as 8 weeks of age, with their number and size increasing over time. The time course of their development was quantified in parallel with analysis of the changes in gene expression of genes associated with ELS formation as well as ocular surface changes associated with development of dry eye disease. Understanding the time course of disease progression and the contribution of LG ELS to disease may improve our understanding of disease mechanisms and identify new therapeutic targets.

## Materials and methods

### Animals

NOR/LtJ (002050) and BALB/cJ (000651) mice were obtained from The Jackson Laboratory (Bar Harbor, ME, USA) and were bred in the vivarium facilities at the University of Southern California. All animal use and experiments were performed in compliance with the University of Southern California Institutional Animal Care and Use Committee (IACUC), and in accordance with the ARVO statement for Animal Use in Ophthalmic and Vision Research and ARRIVE guidelines (ARRIVE 2.0). Components of ELS formation in male NOR mice were evaluated in mice aged 6, 8, 12, 16, 20, and 24 weeks. Initial analysis of ELS formation and changes in ELS-associated gene expression in submandibular gland (SMG) in female NOR mice was also conducted in mice aged 8, 16, 24, and 32 weeks. For analysis of changes in conjunctival goblet cell density, male NOR mice were aged up to 45 weeks. Age-matched male BALB/c mice served as healthy controls in some assays. At ages of interest, mice were anesthetized by intraperitoneal (i.p.) injection with ketamine/xylazine (80–100 mg/kg/5–10 mg/kg) prior to serum collection by cardiac puncture. Mice were then euthanized by cervical dislocation prior to removal of the LG, SMG or other tissue (eyeballs) for specified analysis. A total of 191 male mice (142 male NOR mice, 24 female NOR mice and 25 male BALB/c mice) were used across all experiments. For male NOR mice, use was as follows: 6 weeks, 9 mice; 8–13 weeks, 47 mice; 16 weeks, 30 mice; 20 weeks, 15 mice; 24–35 weeks, 38 mice; and 36–45 weeks, 3 mice. For female NOR mice, use was as follows: 8 weeks, 6 mice; 16 weeks, 6 mice; 24 weeks, 6 mice; 32 weeks, 6 mice. For male BALB/c mice, use was as follows: 8–13 weeks, 14 mice; 16 weeks, 3 mice; 24–35 weeks, 5 mice; and 36–45 weeks, 3 mice. The number of total male mice used per experiment was as follows: H&E staining of LG in paraffin blocks (n=9 mice), comparative H&E staining and immunofluorescence labeling of LG in OCT blocks (n=9 mice), immunofluorescence in tissue sections (n=54 mice), Western blotting (n=15 mice), real time qPCR (n=66 mice), histological quantification of conjunctival goblet cell density (n=26 mice), Luminex ELISA for detection of local cytokines and chemokines (n=24 mice) and ELISA for detection of systemic chemokines (n=12 mice).

### Histological assessment

LG from NOR mice (n=3 LG from 3 mice/group) were collected, fixed in 10% phosphate-buffered formalin, and embedded in paraffin. The LG was sectioned at 5 μm and sections were mounted onto microscope slides. Slides were de-paraffinized by immersing in xylene, dehydrated in ethanol, then stained with hematoxylin-eosin (H&E) according to standard procedures. A separate set of LG sections from optimal cutting temperature (OCT) blocks were also stained with H&E and compared to consecutive LG sections labeled for immunofluorescence (n=3 LG from 3 mice/group) as described in the following section. Tissue sections were imaged using a ZEISS Axioscan 7 Microscope Slide Scanner.

### Immunofluorescence labeling and imaging of ELS components

For IF labeling of components of ELS, LG (n=6 LG from 6 mice/group) were fixed in 4% paraformaldehyde and 4% sucrose in phosphate-buffered saline (PBS) for 3 h at room temperature, followed by incubation in 30% sucrose in PBS overnight at 4 °C. Fixed glands were embedded in OCT blocks, sectioned at 5 or 10 μm, and placed on Superfrost plus^®^ slides. Slides were permeabilized with 0.3% Triton X-100 (Catalogue# T1105, Teknova, Hollister, CA, USA) in PBS (Catalogue#18912014, ThermoFisher Scientific, Waltham, MA, USA) followed by blocking with 5% bovine serum albumin (BSA) (Catalogue# A3912, Millipore Sigma, Burlington, MA, USA) in 0.3% Triton X-100 (in PBS). Tissues were incubated with primary antibodies overnight at 4 °C followed by washing and subsequent addition of appropriate secondary antibodies with incubation for 1 h at 37 °C before washing and mounting. Whole section images were acquired using a ZEISS Axioscan 7 Microscope Slide Scanner.

For analysis of apparent ELS occupancy as a percentage of total tissue area, three tissue sections (10 µm each) taken at 25%, 50%, and 75% depth were analyzed and averaged for each LG. Using B and T cell IF, we identified aggregates characterized by separate regions comprised of B and T cell zones and aggregates that were not segregated into distinct B and T cell zones. The segregation of B and T cells into distinct zones is a characteristic of ELS. QuPath (version 0.5.1) was used to determine and quantify the area occupied by apparent ELS in every tissue section. Briefly, whole tissue images were loaded, and the ‘Magic Wand’ tool was used manually to annotate regions of interest occupied by segregated B/T cells (**Supplementary Figure 1**, Inset 1). This semi-automated selection tool outlines areas based on their signal intensity. Immune cell-enriched areas lacking either B or T cells (**Supplementary Figure 1**, Inset 2) were not included in this quantification of apparent ELS. Finally, the total LG section was also annotated manually to measure the total area/section. The total area occupied by B/T cells in apparent ELS was expressed as a percentage of total tissue area.

The total IF signal occupied by B and/or T cells was also quantified as the area occupied by a positive IF signal, also expressed as a percentage of the total tissue area, using ImageJ 1.54. This measurement considered all signals, including apparent ELS, separate B and T cell infiltrates, and individual immune cells dispersed throughout the LG. The positive signal was defined by setting a lower threshold of 100 for each image based on signal-to-background contrast, which was consistently applied across all images. The percentage of the area occupied by the positive signal was calculated by dividing the area of the positive signal above the defined threshold by the total tissue section area, then multiplying by 100.

In addition to analysis of B and T cell infiltration by IF, additional markers were visualized by IF in the LG, including those for HEV, FDC, plasma cells, GL7+ germinal center B cells, and IgG. Tissue sections of 5 µm thickness were used for labeling these markers, and confocal fluorescence microscopy with a Zeiss 800 LSM equipped with Airyscan (Zeiss, Thornwood, NY, USA) was used for acquisition of higher magnification images. Primary and secondary antibodies used for analysis of ELS components are included in **Supplementary Table 1**. A comparable qualitative analysis of B and T cell infiltration by IF was conducted in SMG of female NOR mice aged 8, 16, 24 and 32 weeks of age (n=6 SMG from 6 mice/group).

### Western Blotting of LG lysates

To detect the relative abundance of IgG antibodies associated with ELS formation, LG (n=3 LG from 3 mice per group) were collected from male NOR mice at pre-disease (6 weeks), early disease (8 weeks), intermediate disease (16 weeks) and established disease (24 weeks) and also from healthy control BALB/c mouse LG (8 weeks). Lysates were prepared by homogenizing a single LG per mouse in BeadBug prefilled tubes (Catalog# Z763780, Millipore Sigma, Burlington, MA, USA) containing 0.1% Triton X-100 diluted in PBS and protease inhibitor cocktail (Catalog# 5872S, Cell Signaling Technology, Danvers, MA, USA). Tissue homogenates were centrifuged at 8000 x g for 10 min at 4 °C, and supernatant was collected and re-centrifuged using the same conditions. Protein concentration was measured using the Pierce BCA Protein Assay Kit (Catalog# 23275, ThermoFisher Scientific, Waltham, MA, USA). Lysates were incubated with Laemmli sample buffer (Catalogue #1610747, BioRad, Irvine, CA, USA) and β-mercaptoethanol (Catalogue #1610710, BioRad, Irvine, CA, USA) at 100 °C for 10 min. 40 µg of protein from each sample was loaded on precast 10% Tris-Glycine gels and resolved by SDS-PAGE (Catalogue # XP00105BOX, ThermoFisher Scientific, Waltham, MA, USA) at 90 V at 4 °C and transferred to nitrocellulose membranes (Catalogue #IB23001, ThermoFisher Scientific, Waltham, MA, USA) using an iBlot^®^ 2 gel transfer device (Life Technologies, Carlsbad, CA, USA). The total protein in each lane was measured using the Revert 700 Total Protein Stain Kit for normalization of protein loading, and the signal was read using the LICOR Odyssey Fc imager. After destaining, the membrane was blocked using 5% BSA in Tris buffered saline with Tween 20 (TBS-T) (Catalogue #T9039, Millipore Sigma, Burlington, MA, USA) for 1 h at room temperature followed by incubation with a polyclonal Donkey anti-mouse IgG antibody (Catalogue# 926-68072, LICORbio, Lincoln, NE, USA) (**Supplementary Table 1**) diluted 1:2000 in blocking buffer for 1 h at room temperature followed by three washes for 5 min each with TBS-T. The membrane was then imaged using a LI-COR Odyssey Fc imager. Image Studio software version 5.2 was used for quantification of signal intensity.

### RNA extraction and quantitative reverse transcription PCR

ELS initiation and development is associated with temporal changes in local gene expression. Changes in expression of genes of interest in the LG was conducted in a pre-diseased mouse group lacking lymphocytic infiltration (6 weeks) as well as at 4-week intervals from 8–24 weeks when lymphocytic infiltration of the LG was readily detected (n=6–9 LG from 6–9 mice/group). Total RNA from the LG of male NOR mice at the indicated ages was extracted using the RNeasy plus Universal Mini Kit (Catalog # 73404, Qiagen, Hilden, Germany) according to the manufacturer’s instructions. The concentration and purity of RNA was assessed using a Nanodrop OneC spectrophotometer. cDNA templates were synthesized from 4 µg RNA using TaqMan^®^ gene expression assays for the qPCR reaction. RT-qPCR was performed using TaqMan minor groove binder probes as listed in **Supplementary Table 2**, on a QuantStudio 6 Flex machine. Gene expression analysis was performed using the 2^−ΔΔCt^ method. Changes in gene expression levels are presented as log_10_ fold changes relative to control (log_10_ 2^–ΔΔCt^). Additional studies probed changes in a subset of these genes in **Supplementary Table 2** in SMG isolated from female NOR mice at 8, 16, 24 and 32 weeks (n=6 SMG from 6 mice/group).

### Detection of local and systemic cytokines and chemokines

Concentrations of LG cytokines and chemokines were determined in LG lysates using the 36-Plex Discovery assay array performed by Eve Technologies (Calgary, AB, Canada). Lysates were prepared as described above for Western Blotting. Radioimmunoprecipitation assay buffer (RIPA) buffer and protease inhibitor cocktail (Catalog# 5872S, Cell Signaling Technology, Danvers, MA, USA) were used as the lysis buffer. Protein concentration was measured using Pierce BCA Protein Assay Kit (Catalog# 23275, ThermoFisher Scientific, Waltham, MA, USA) followed by Luminex assay. The sample size was 6 LG from 6 mice per group, and all samples and standards were analyzed in duplicate. For detection of systemic CXCL13, blood from NOR mice aged 8, 16, and 24 weeks was collected by cardiac puncture. Blood from healthy control BALB/c mice age-matched to the intermediate NOR disease at 16 weeks was also included. Blood was allowed to clot for 2 h at room temperature and centrifuged at 1500 x g for 15 min at 4 °C. Serum was collected from the supernatant and stored at −80 °C until use. CXCL13 was quantified in serum diluted four-fold using a commercial ELISA kit (Catalog# MCX130, R&D Systems, Minneapolis, MN, USA) (n=3 mice per group), with all samples and standards analyzed in duplicate. Absorbance was measured at 450 nm with a wavelength correction at 570 nm using a SpectraMax iD3 microplate reader (Molecular Devices, San Jose, CA, USA).

### Conjunctival goblet cell quantification

Goblet cell density was determined in male NOR mice at early (8–13 weeks) and established/advanced (24–35 weeks) disease. An older cohort (36–45 weeks) was added to fully capture onset and progression of ocular surface disease which was delayed relative to the time course of LG infiltration (n=3–5 mice per group). Mice were euthanized as previously described, and eyeballs, along with the eyelids, were isolated, fixed in 10% formalin, and embedded in paraffin. Sectioning was performed across the entire eyeball and eyelids to map the distribution of goblet cells. Tissue sections were stained with Periodic Acid Schiff (PAS) reagent, and goblet cell density was measured using NIS-Elements software (AR, version 5.20.2; Nikon Melville, NY, USA) and expressed as the number of positive cells per millimeter of conjunctiva per eye (18).

Statistical analysis: Statistical analyses were performed using GraphPad Prism software version 8.4.3 (GraphPad, San Diego, CA, USA). Differences between three or more groups were analyzed using a one-way ANOVA test followed by Tukey’s multiple comparisons. Comparison between two mouse strains at different age groups for conjunctival goblet cell density analysis was performed using a two-way ANOVA test followed by Sidak multiple comparison test using GraphPad Prism software version 10.6.1 (GraphPad, San Diego, CA, USA).

## Results

### Histological evidence of zonal organization within foci in the LG

**Figure 1:**
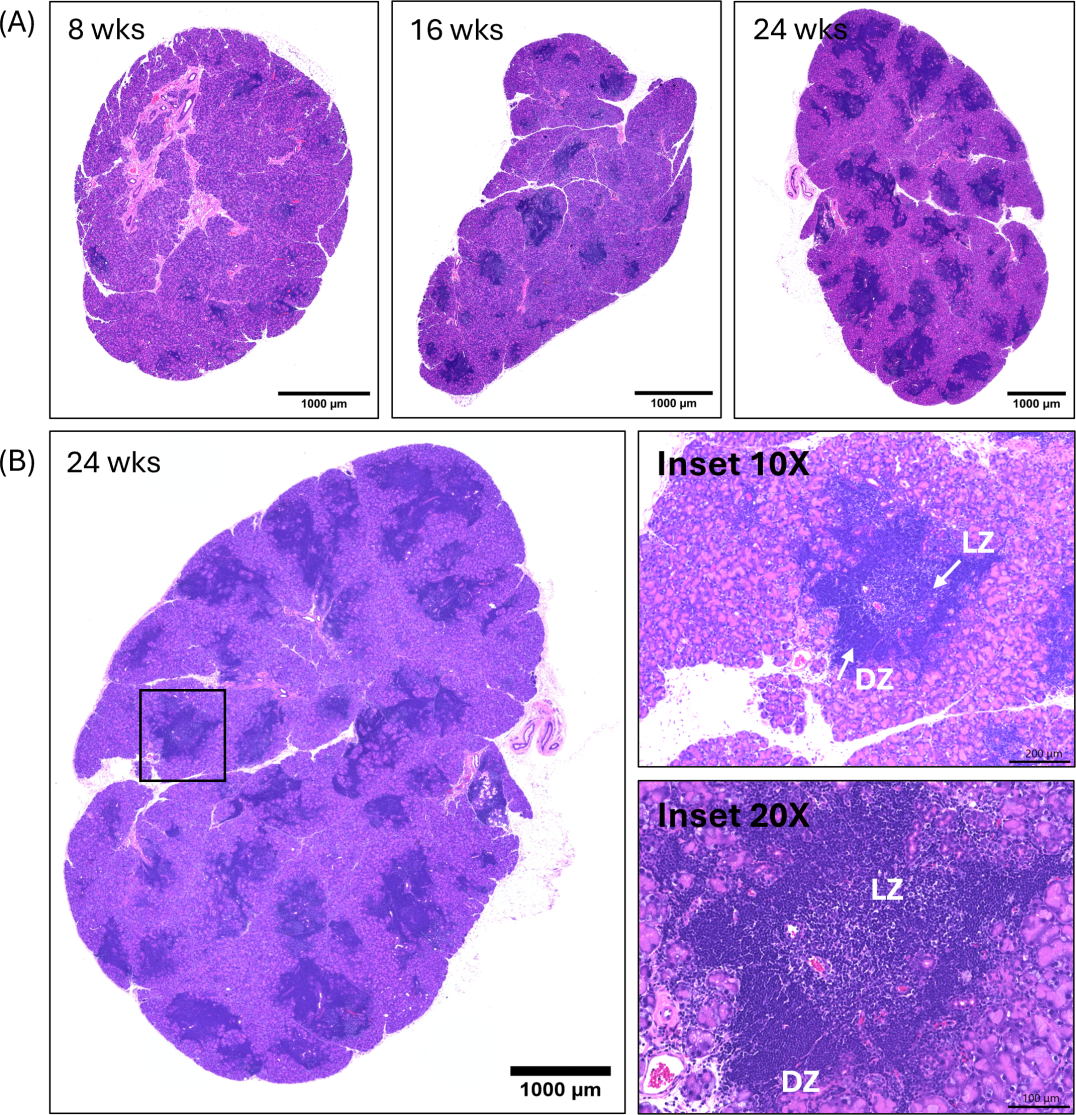
Representative images showing lymphocytic infiltration in LG of male NOR mice. **(A)** H&E staining of LG sections at early (8 weeks), intermediate (16 weeks) and established (24 weeks) disease that were fixed and sectioned in paraffin blocks. Scale bar, 1000 μm. **(B)** Representative image showing lymphocytic infiltration in established disease (24 weeks). Scale bar 1000 μm. The boxed regions in **(B)** are shown in the Inset panels. Insets show magnification at 10X (Scale bar, 200 μm) and 20X (scale bar, 100 μm), showing compartmentalization of foci into apparent dark zones (DZ) and light zones (LZ). N = 3 LG from 3 mice per age group.

The presence and abundance of lymphocytic infiltrates with properties of ELS was first analyzed in LG from male NOR mice using H&E staining (**Figure 1A**), and results used to determine the age range of interest for analysis of subsequent features of ELS in LG. Lymphocytic infiltrates appeared in the LG as early as 8 weeks of age (early LG disease), increased in size and number by 16 weeks of age (intermediate LG disease) and further increased in size and number to occupy a substantial portion of the LG by 24 weeks of age (established LG disease). Germinal centers within SLO are detectable histologically by formation of distinct dark (DZ) and light (LZ) zones (19). As shown in **Figure 1B**, LG infiltrates in 24-week male NOR mice showed identifiable DZ and LZ. Comparable germinal center organization within infiltrates was also detectable in LG sections at early and intermediate disease (**Supplementary Figure 2**), suggesting that a range of 8–24 weeks was appropriate for characterizing the time course of formation and features of LG ELS in this model.

### ELS accumulation and development of distinctive morphological features increased with age in LG of male NOR mice

ELS, like SLO, exhibit unique morphological features (B/T cell segregation, formation of HEV) and contain specific cell types (FDC), essential for initiating adaptive immune responses. Dual IF labeling of IgD (naïve B cell marker) and CD3 (T cell marker) enabled characterization of B/T cell compartmentalization from early to established LG disease. Clusters of immune cells labeled by IF showed either partial or full segregation of B and T cells (**Supplementary Figure 1**, Inset 1). In the absence of a surrounding capsule, the foci in the LG were diffuse and irregularly-shaped rather than the more organized SLO; however, distinct segregation of B and T cells was observed in most infiltrates. Occasional clusters of cells that did not show distinct segregation of B/T cells were also detected (**Supplementary Figure 1**, Inset 2).

**Figure 2:**
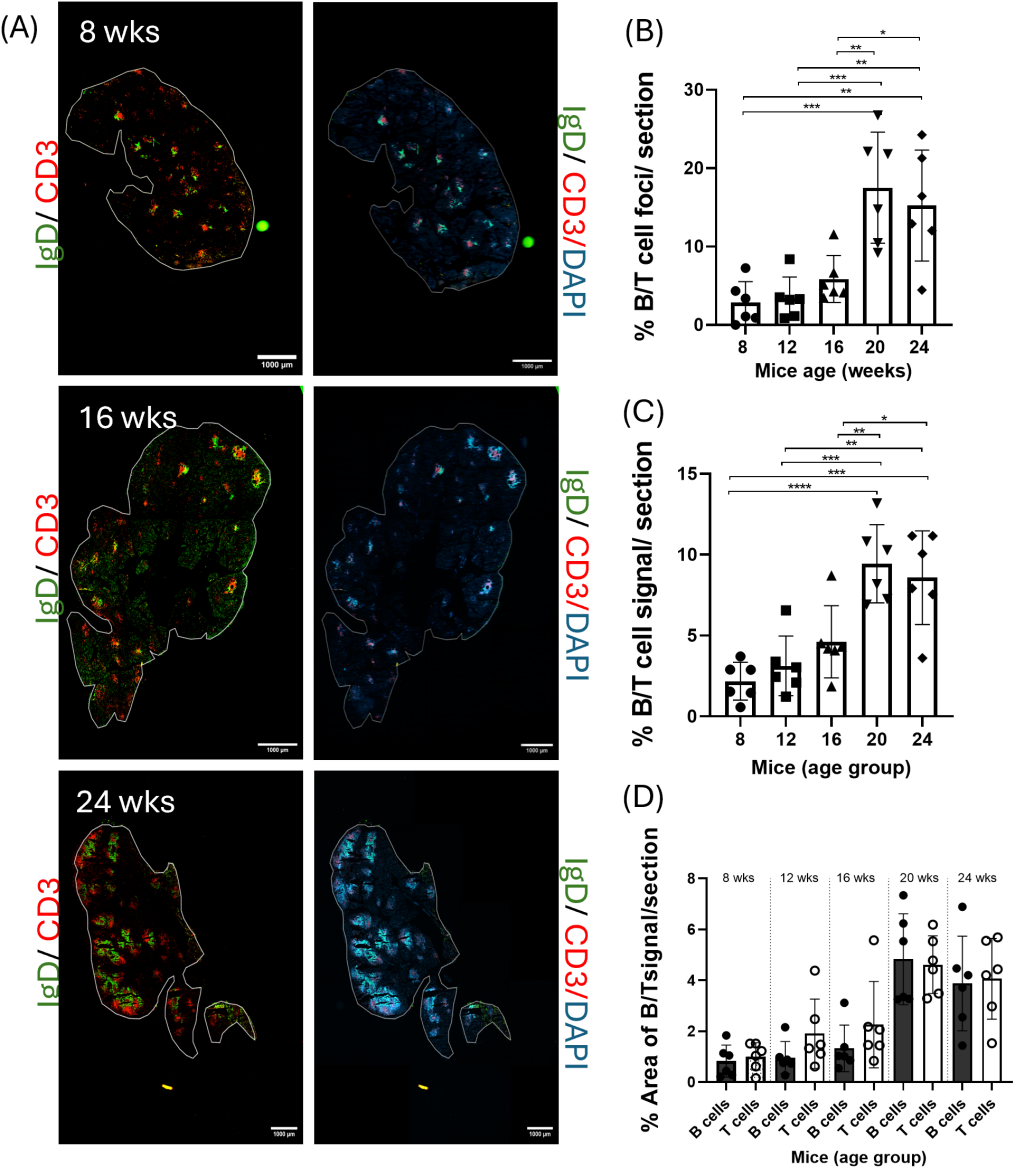
B/T cell aggregates in LG of male mice at early (8 weeks), intermediate (16 weeks) and advanced (24 weeks) disease. **(A)** Representative IF images show components of B/T cell aggregates using antibodies to IgD, highlighting naive B cells (green) and CD3, highlighting T cells (red). DAPI labels nuclei (blue). Scale bar, 1000 μm. **(B)** Graph showing area occupied by B/T cell aggregates as a percentage of the whole section area. **(C)** Graph showing area occupied by B and T cell fluorescent signal as a percentage of the whole section area. **(D)** Graph showing a comparison of the area occupied by B cells versus T cell fluorescence in LG from mice at each age. Data for **(B-D)** are from N = 6 LG from 6 different mice per age group each N representing their compiled average from 3 separate sections cut at 25, 50 and 75% depth of the LG. Results presented as mean ± SD. Statistical significance is indicated as *P<0.05, **P<0.01, ***P<0.001, ****P<0.0001.

The area of the LG occupied by B/T cell aggregates, termed ‘apparent ELS,’ was quantified as a percentage of occupancy of the total LG area (**Figure 2**). Representative images at early, intermediate and established disease are shown in **Figure 2A**. Organized aggregates were detected in LG from mice as early as 8 weeks of age. In LG of younger mice, apparent ELS were smaller, occupied less tissue area [2.8 ± 2.7% infiltration], and displayed less cellular segregation. ELS occupancy of the LG area increased steadily with age, becoming significantly greater than in early disease by 20 weeks of age [17.5 ± 7.1% infiltration, P-value<0.01] (**Figure 2B**). Apparent ELS also showed an increase in size with age, with adjacent aggregates appearing to merge.

The percentage of LG area occupied by B- and/or T-cell immunofluorescence signal per LG area was also determined (**Figure 2C**), showing a similar increase in the percentage of tissue area occupied as the disease progressed. This increase was significant and also distinguishable from early disease in LG from 20 week mice [% area occupied by B/T cell fluorescence =9.4% ± 2.4, P-value<0.05]. We noted that the % total LG area occupied by apparent ELS was greater than that occupied by B/T cell fluorescence; these clusters were outlined regionally and manually, thus including ELS with regions that did not display fluorescence, as illustrated in **Supplementary Figure 1**, inset 1. There was no significant differences in the ratio of B:T cells in LG infiltrates over time (**Figure 2D**).

**Figure 3:**
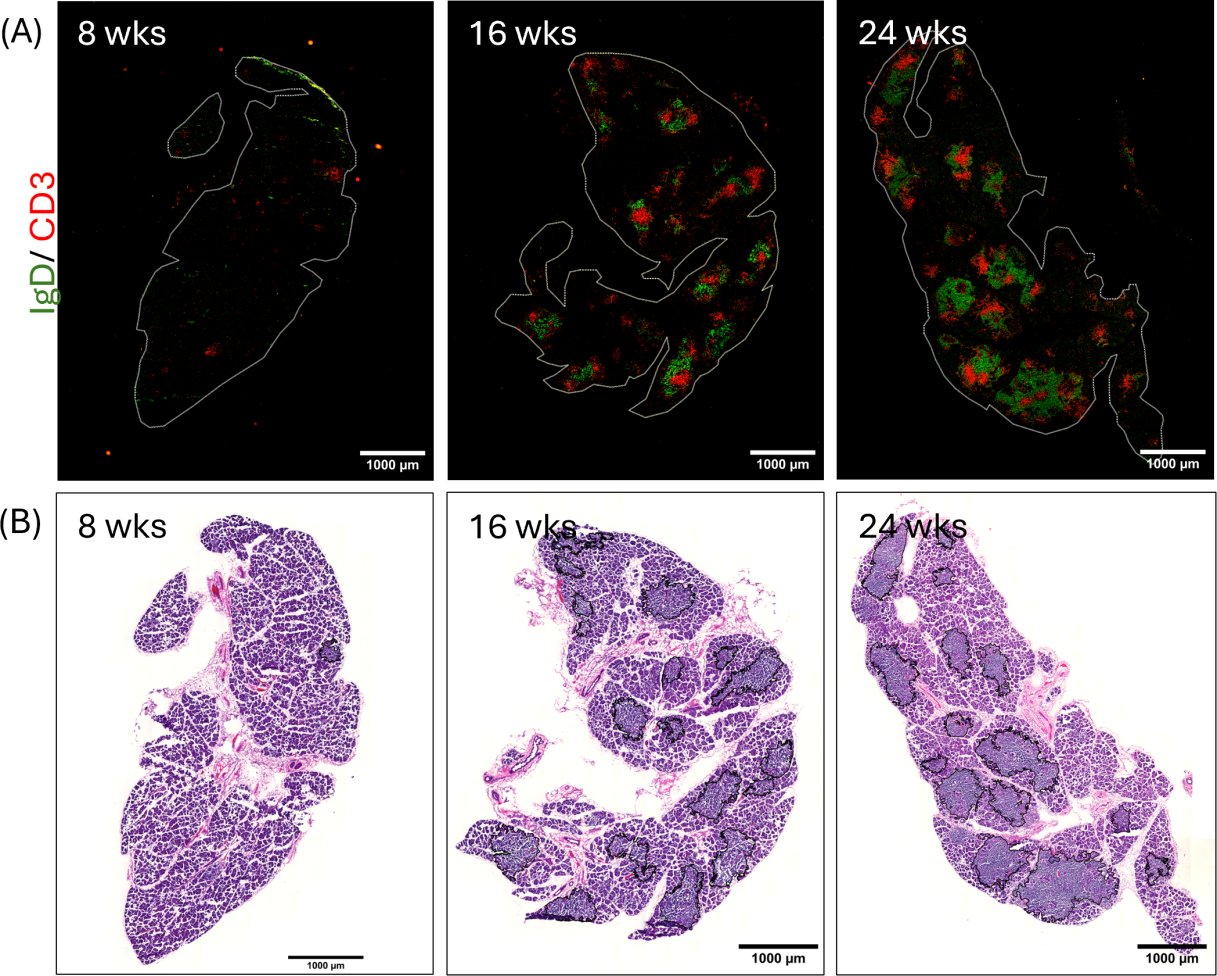
Representative images showing lymphocytic infiltration of LG from male NOR mice at early (8 week), intermediate (16 week), and advanced (24 week) disease stages. Sequential sections of LG were cut at 5 μm thickness from tissue in O.C.T. Panel **(A)** shows sections from each LG labeled with primary and fluorescent secondary antibodies to IgD (naive B-cells, green) and CD3 (T cells, red). Panel **(B)** shows H&E staining of the sequential section from the same LG. Scale bar, 1000 μm. N = 3 LG from 3 different mice per age group.

H&E has historically been used to quantify the extent of lymphocytic infiltration in LG and SG in patient biopsies and in tissue from SjD animal models; however, it provides limited information about the nature of the cells within the infiltrates. To compare the labeling patterns and information obtained from H&E versus IF analysis of LG tissue infiltration, consecutive sections of LG from male NOR mice with early, intermediate and established disease were prepared from OCT blocks, and labeled by H&E and for B/T cell IF. Despite the lesser quality of the histological specimens obtained from OCT blocks, there was a clear morphological correlation between consecutive sections. Thus, areas of immune cell infiltration labeled for B/T cell IF (**Figure 3A**) were comparable to areas identified by H&E staining (outlined in black-**Figure 3B**). IF labeling was subsequently used to provide information regarding the identity and organization of other cells in apparent ELS.

### Apparent ELS in NOR mouse LG exhibit HEV and FDC

**Figure 4:**
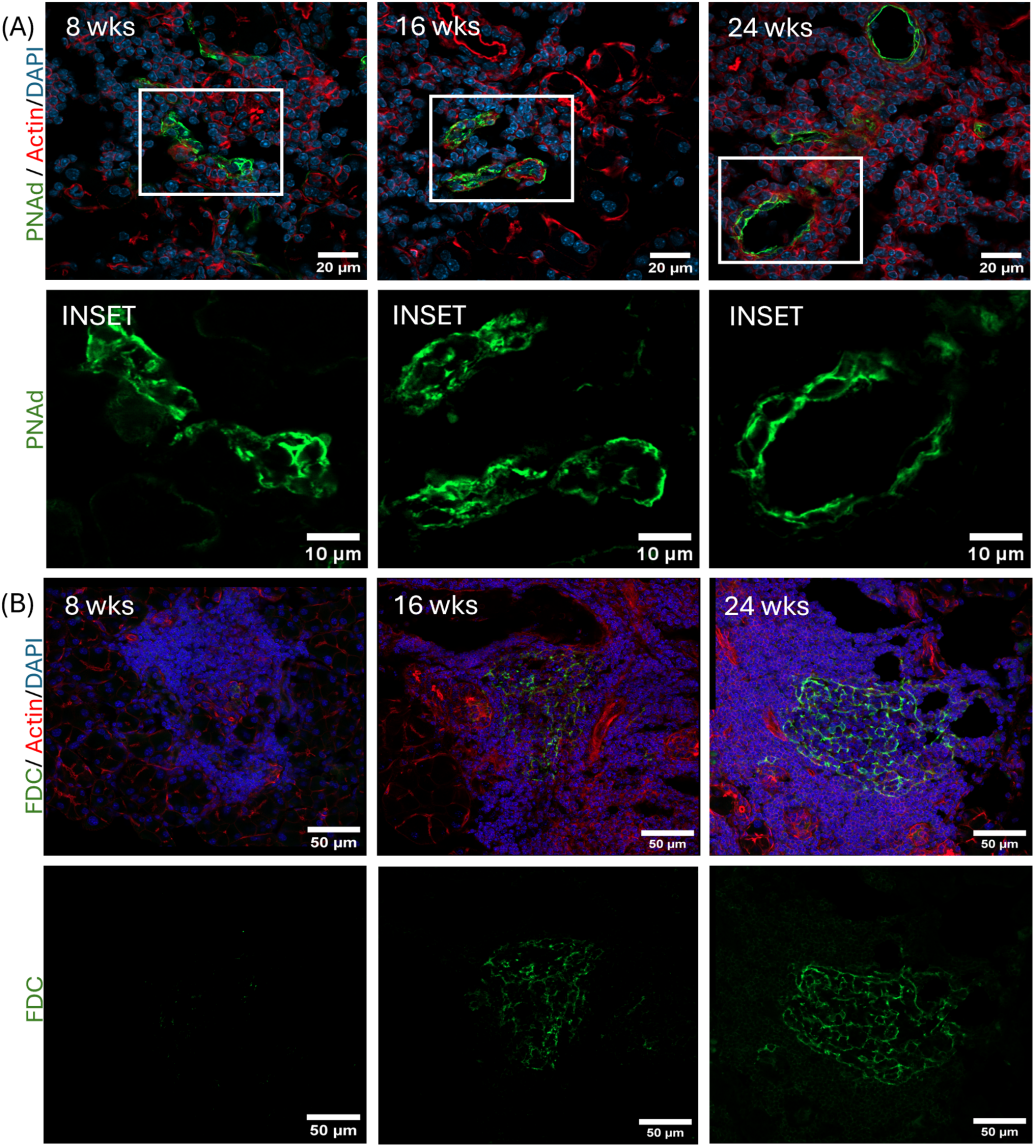
Representative IF images from male NOR mouse LG showing ELS-associated markers at the indicated mouse ages. **(A)** High endothelial venules in LG were labeled with antibody to PNAd (green) while rhodamine phalloidin and DAPI labeled actin (red) and nuclei (blue), respectively. Scale bar, 20 µm. Boxed regions in each image are shown in the Inset panels below. Scale bar in insets, 10 um. **(B)** Follicular dendritic cells in LG sections at the same indicated ages were labeled with antibodies to CD21/CD35 (green) while rhodamine phalloidin and DAPI labeled actin (red) and nuclei (blue), respectively. Scale bar= 50 µm. The bottom panel shows the FDC IF (green) alone to visualize the morphology of FDC. Scale bar= 50 um. N = 3 LG from 3 mice per age group.

To further test whether LG infiltrates showing segregated B/T cells were ELS, HEV and FDC were investigated using IF. As shown in **Figure 4A**, small HEV were detectable in immune cell infiltrates at the earliest disease stage, and evolved into larger and wider venules as disease developed. Insets show PNAd IF at higher magnification. Also characteristic of SLO, FDCs form an organized follicular structure which promotes antigen-driven selection of B cells. High levels of complement receptor 1 (CD35) and 2 (CD21) are expressed in FDCs enabling antigen retention and high-affinity B cell maturation. **Figure 4B** demonstrates the presence of an FDC network detectable in infiltrates in LG as early as 16 weeks of age, becoming more prominent at 24 weeks. Collectively, these findings support our assertion that ELS form early in LG disease and evolve with disease progression.

**Figure 5:**
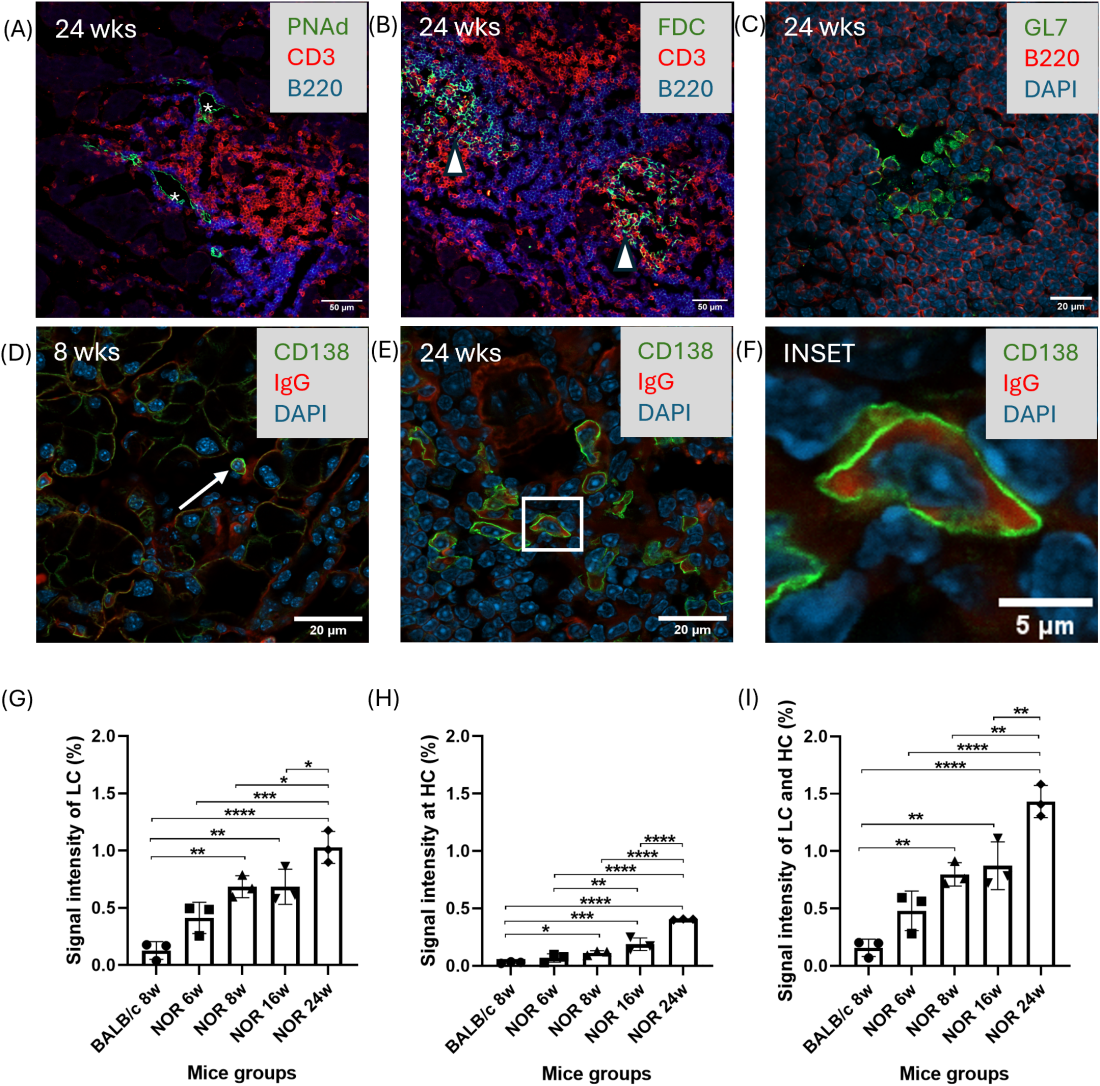
Representative immunofluorescence images of ELS components in male NOR mouse LG and evidence of increase of increased total tissue IgG. **(A)** High endothelial venules (shown in asterisk*) at the B/T cell border using antibodies to PNAd+ (green), CD3+ T cells (red) and B220+ B cells (blue) in LG from 8 week mice. Scale bar=50 µm. **(B)** Follicular dendritic cells (shown in arrowhead) present within a B cell-enriched zone (blue) interspersed with scattered T-cells (red) in LG from 24 week mice. Scale bar=50 µm. **(C)** Presence of GL7+ B cells indicative of functional B cell activation within a B cell enriched region using antibodies against GL7 (green), B220 (red) and DAPI (nuclei). Scale bar=20 µm. **(D)** CD138+ plasma cell immunofluorescence (green) overlaps with IgG (red) indicating local production/storage in LG sections from 8 week mice (shown in arrow) and **(E)** 24-week mice, showing the increasing abundance of plasma cells in ELS in established disease, DAPI labels nuclei, scale bar=20 µm. The boxed region in the 24-week panel is expanded in the inset **(F)**, scale bars in inset=5 μm. N=3 LG from 3 mice per age group. **(G-I)** Light chain (LC), Heavy chain (HC) and total (LC+HC) signals from Western blotting measuring changes in IgG levels in male BALB/c and NOR mouse LG lysates at the indicated mouse ages **(G)** LC IgG signal intensity in lysates was quantified at 25KDa, **(H)** HC IgG signal intensity was quantified at 50KDa, and **(I)** Total IgG signal (LC+HC) signal. N=3 LG from 3 mice per age group. Lysates were prepared and analyzed for Western blotting using a secondary donkey anti-mouse antibody conjugated to IRdye 680 as described in Methods. Results are presented as mean ± SD. Statistical significance is indicated as *P<0.05, **P<0.01, ***P<0.001,****P<0.0001.

To elucidate the spatial distribution of ELS-associated markers relative to B and T cell aggregates, LG with established disease at 24 weeks were further characterized. HEV developed at T-B cell borders (**Figure 5A**). In contrast, FDC were primarily detected within B cell-enriched regions, as expected since these cells are essential for B-cell activation, with some scattered CD3+ cells also found within the FDC network (**Figure 5B**). GL7+ germinal center B cells were also identified within lymphocytic infiltates (**Figure 5C**).

To better understand the autoimmune features of ELS, plasma cells within ELS were labeled to detect IgG. Elevated levels of IgG in SjD are reported, while anti-SSA and anti-SSB, hallmarks of the disease, are also IgG (20). **Figure 5D** shows that occasional plasma cells (CD138+) were present in LG from NOR mice at 8 weeks of age. These plasma cells were more abundant, localized within lymphocytic infiltrates, and extensively colocalized with IgG in LG of 24 week NOR mice, suggesting active local production or storage of IgG (**Figures 5E, F**).

To confirm the apparent increase in IgG IF seen in male NOR mice, IgG heavy chain (HC) and light chain (LC) content of LG lysates were determined using western blotting with a secondary antibody to mouse IgG. As shown in **Supplementary Figures 3A**, a progressive increase in the signal intensity of bands at 25KDa (LC) and 50KDa (HC) were seen in lysates from male NOR mouse LG while minimal IgG signal was detected in BALB/c mouse LG lysates. Densitometric quantification using total protein for normalization (**Supplementary Figure 3B**) showed significantly increased LC (**Figure 5G**), HC (**Figure 5H**) and combined IgG signal (**Figure 5I**) in male NOR mouse LG lysates relative to levels in healthy control BALB/c. IgG values also rose significantly between 8 weeks of age and 24 weeks of age with disease progression in male NOR mouse LG.

### Ectopic expression of genes associated with ELS formation in NOR mice increased as disease progressed

**Figure 6:**
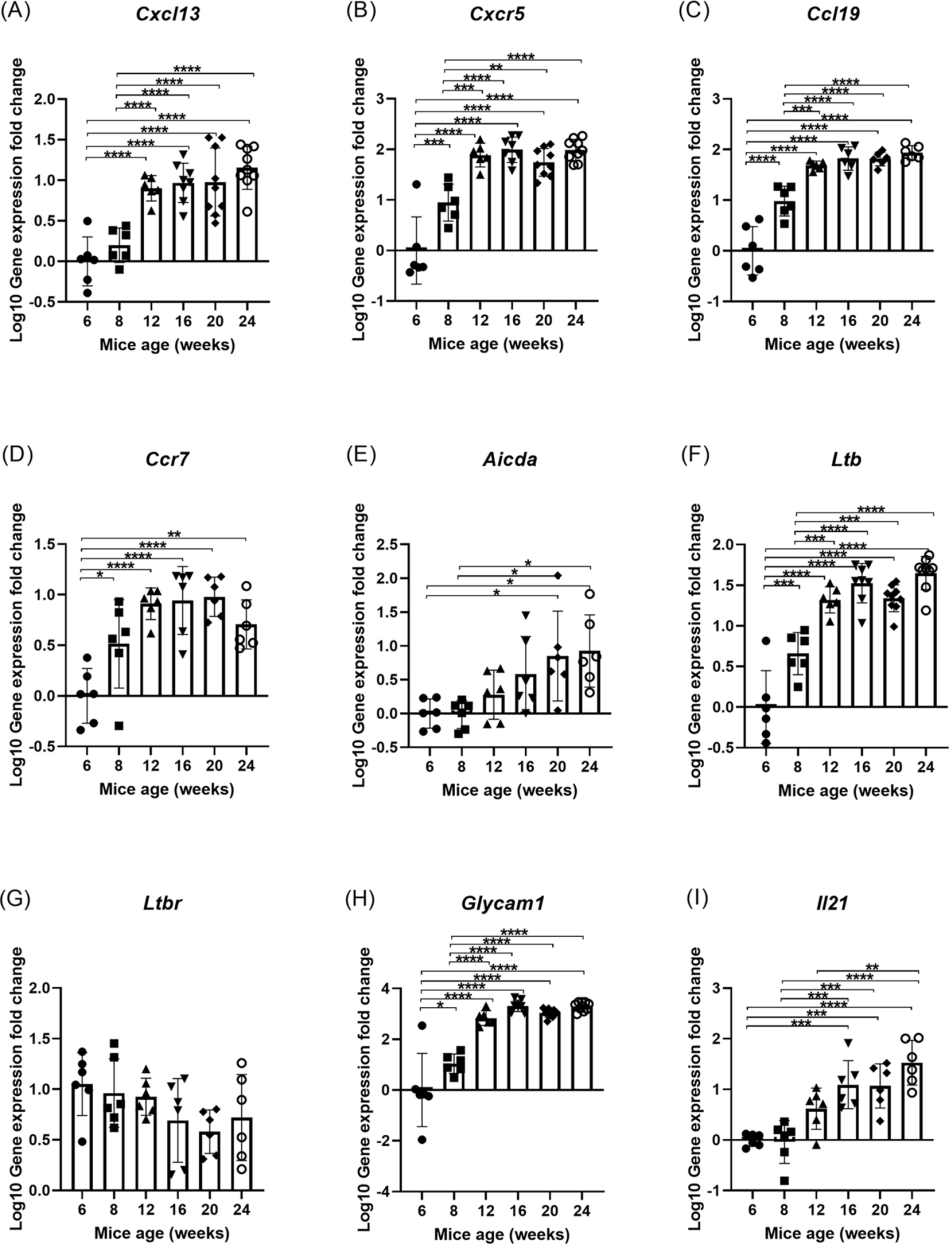
RT-qPCR analysis of mRNA expression of genes related to ELS development in the LG of male NOR mice at different ages. **(A)** Cxcl13, chemotactic for subsets of B and T cells; **(B)** Cxcr5, chemokine receptor for CXCL13; **(C)** Ccl19, chemoattractant for naïve T cells; **(D)** Ccr7, chemokine receptor for CCL19; **(E)** Aicda, activation-induced cytidine deaminase (AID) enzyme involved in somatic hypermutation of B cells; **(F)** Ltb, involved in the formation of lymphoid tissue; **(G)** Ltbr, receptor for LTB; **(H)** Glycam1, High endothelial venule marker; and **(I)** Il21, cytokine produced by TFH cells involved in B cell activation and differentiation. N = 6–9 LG from 6–9 mice per age group, with each data point representing one LG from one mouse. Results presented as mean ± SD. Statistical significance is indicated as *P<0.05, **P<0.01, ***P<0.001,****P<0.0001.

LG expression of genes associated with the initiation, development and maintenance of ELS was also analyzed. B cell survival, proliferation, and function within ELS requires specific chemokines and their receptors, such as CXCL13/CXCR5 and CCR7/CCL19. CXCL13 is chemotactic for B cells and secreted by FDCs and T-follicular helper (TFH) cells to direct B cells expressing CXCR5 to lymphoid follicles. Elevated *Cxcl13* levels were observed in the NOR mouse LG as early as 12 weeks of age and remained elevated through 24 weeks of age (**Figure 6A**). A significant increase in gene expression of *Cxcr5* occurred even earlier, by 8 weeks of age (**Figure 6B**). A significant elevation in gene expression of *Ccl19* and *Ccr7* was also seen by 8 weeks of age (**Figures 6C, D**) and remained elevated through 24 weeks. Activation-induced cytidine deaminase (AID) is critical for B cells undergoing somatic hypermutation and class switch recombination (21). *Aicda* gene expression showed an upward trend in expression that became statistically significant by 20 weeks (**Figure 6E**). Significant upregulation in expression of *Ltb* (**Figure 6F**), critical for induction of lymphoid cytokines, was detected by 8 weeks of age. However gene expression of its receptor, *Ltbr*, was not increased up to 24 weeks of age (**Figure 6G**). Gene expression of *Glycam1* was significantly upregulated by 8 weeks of age (**Figure 6H**), consistent with the early detection of HEV by IF. IL21, primarily produced by TFH cells, is linked to B cell activation and differentiation (22), and its gene expression also increased steadily from 16 weeks of age (**Figure 6I**).

**Figure 7:**
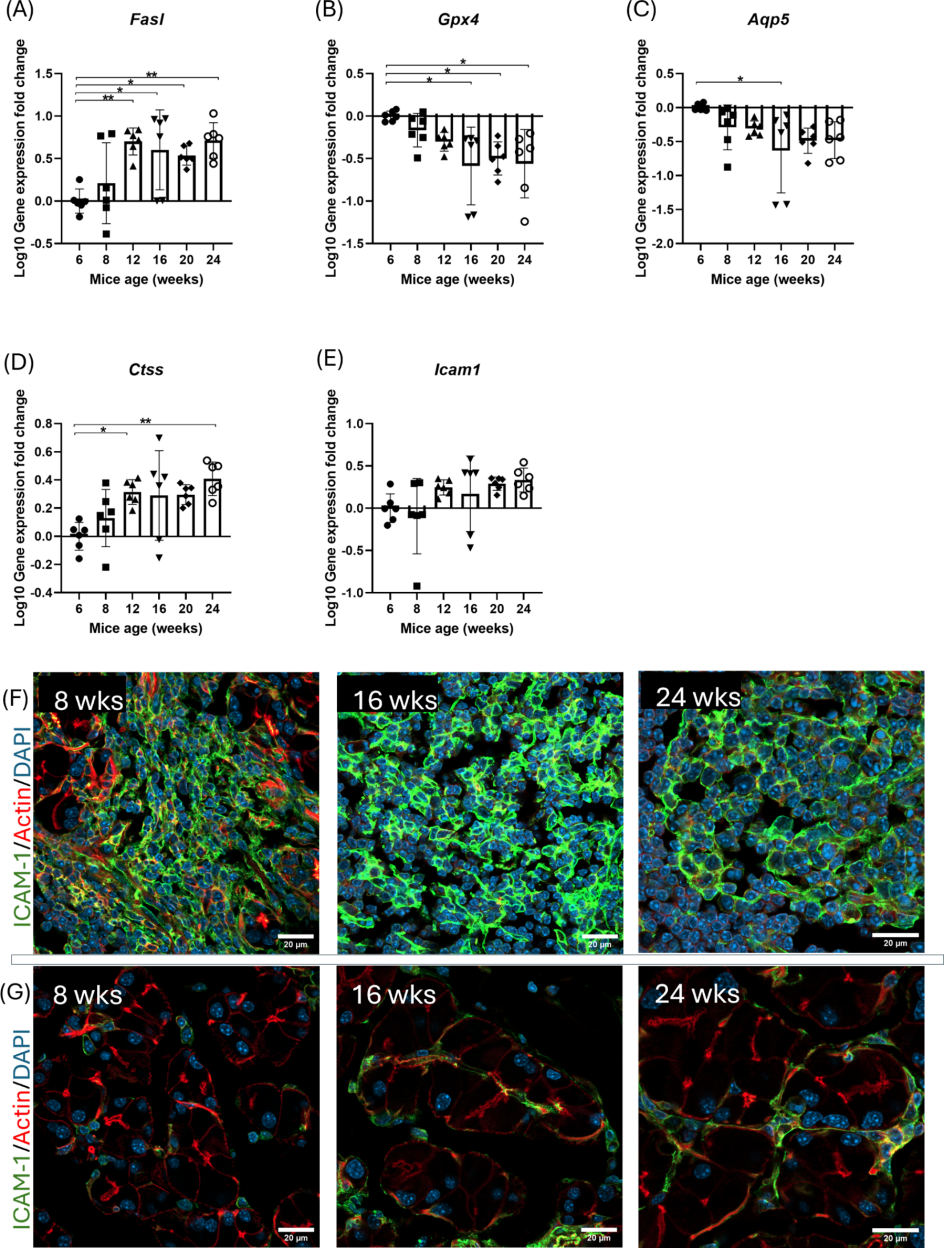
Changes in gene expression of genes related to overall glandular health and function. RT-qPCR analysis of mRNA expression of genes associated with: **(A)** cellular apoptosis (*Fasl*), **(B)** oxidative stress and ferroptosis (*Gpx4*), **(C)** glandular secretory function (*Aqp5*), **(D)** inflammation (*Ctss*), **(E)**, and cellular trafficking of immune cells (*Icam1*); at different ages, N = 6 LG from 6 mice per age group. Results presented as mean ± SD. Statistical significance is indicated as *P<0.05, **P<0.01. Representative immunofluorescence images of ICAM expression in early, intermediate and established disease in **(F)** ELS and **(G)** acinar cells. ICAM-1 IF shown in (green), Rhodamine phalloidin and DAPI label actin (red) and nuclei (blue). Scale bar, 20 µm. N = 3 LG from 3 mice per age group.

To gain insights into the time course of ELS formation with respect to changes in other LG markers related to overall glandular health, expression profiles associated with changes in secretory function, apoptosis, oxidative stress and autoimmune epithelitis were determined. Dysregulated glandular homeostasis associated with acinar cell apoptosis precedes and triggers lymphocytic infiltration of the LG (23). A significant upregulation of *Fasl*, associated with LG apoptosis (24), was seen by 12 weeks of age and remained elevated through 24 weeks of age (**Figure 7A**). Additionally, a significant reduction in gene expression of glutathione peroxidase 4 (*Gpx4*), which suppresses ferroptosis (25), was detected by 16 weeks of age (**Figure 7B**). In the LG, a significant reduction in *Aqp5* gene expression was also seen by 16 weeks of age (**Figure 7C**), possibly reflecting decreased fluid transport associated with the AQP5 water channel. A strong correlation between downregulated GPX4 and reduced aquaporin 5 transcription via STAT4 has been reported in the SG in SjD (25). Cathepsin S (CTSS) is a multifunctional cysteine protease (24) and a potential biomarker of SjD in tears (12). Temporal changes in Cathepsin S (CTSS) gene expression and activity in NOD and NOR murine models of SjD has been reported (10). In NOR mouse LG, *Ctss* was elevated significantly by 12 weeks and remained elevated through 24 weeks of age (**Figure 7D**), extending previous analysis (10). Lastly, intercellular adhesion molecule-1 (ICAM1) is implicated in autoimmune epithelitis (26). While gene expression studies showed only a modest increase in *Icam1* (**Figure 7E**), protein expression in LG tissue sections showed extensive and abundant ICAM-1 expression within lymphocytic infiltrates as early as 8 weeks (**Figure 7F**), consistent with its essential role in immune cell homing (27). ICAM-1 was also detected in other regions including the plasma membrane of acini and within central acinar lumena in LG from NOR mice with intermediate and established disease (**Figure 7G**), consistent with its increase in the LG previously reported in the related NOD model (28).

### NOR mouse LG exhibited early and persistant elevation of some ELS cytokines and chemokines

The protein concentration of several cytokines and chemokines of interest was tested in LG lysates of NOR mice at the three ages corresponding with early, intermediate and established disease stages, in comparison to their levels in LG lysates from young BALB/c mice. Some targets investigated are shown in **Supplementary Figures 4A**, which revealed small changes in pro-inflammatory (TNF, IL-1α) and anti-inflammatory (IL-10) cytokines. In addition, the panel included various chemokines involved in homing of lymphocytes to ELS in target organs such as CCL3, CXCL5, CXCL10 and CCL5 that are elevated either in SjD patient saliva samples and/or in saliva and serum samples from mouse models of SjD (29, 30). Consistent with increased gene expression of early effectors of ELS formation including *Cxcr5*, *Ccl19*, *Ccr7*, and *Ltb*, some of these proteins were also significantly elevated at early disease and remained elevated over time, including TNF, IL-1α, CCL3, CXCL5, and CXCL10. A slight yet significant reduction in anti-inflammatory IL10 was observed in 24 week old NOR mouse LG lysate relative to young BALB/c mice (P-value<0.05).

CXCL13 is an essential chemokine which regulates B cell trafficking and lymphoid structure organization, with levels correlated with disease activity and lymphoma risk (31). Several cell types may produce CXCL13 including FDC, macrophages, dendritic cells and possibly TFH cells (32). Male NOR mouse serum showed comparable CXCL13 levels relative to male BALB/c mouse serum at 8 weeks of age but its levels were significantly elevated in mice with established disease at 24 weeks of age (P-value<0.05) (**Supplementary Figure 4B**).

### LG ELS formation and ocular surface disease

**Figure 8:**
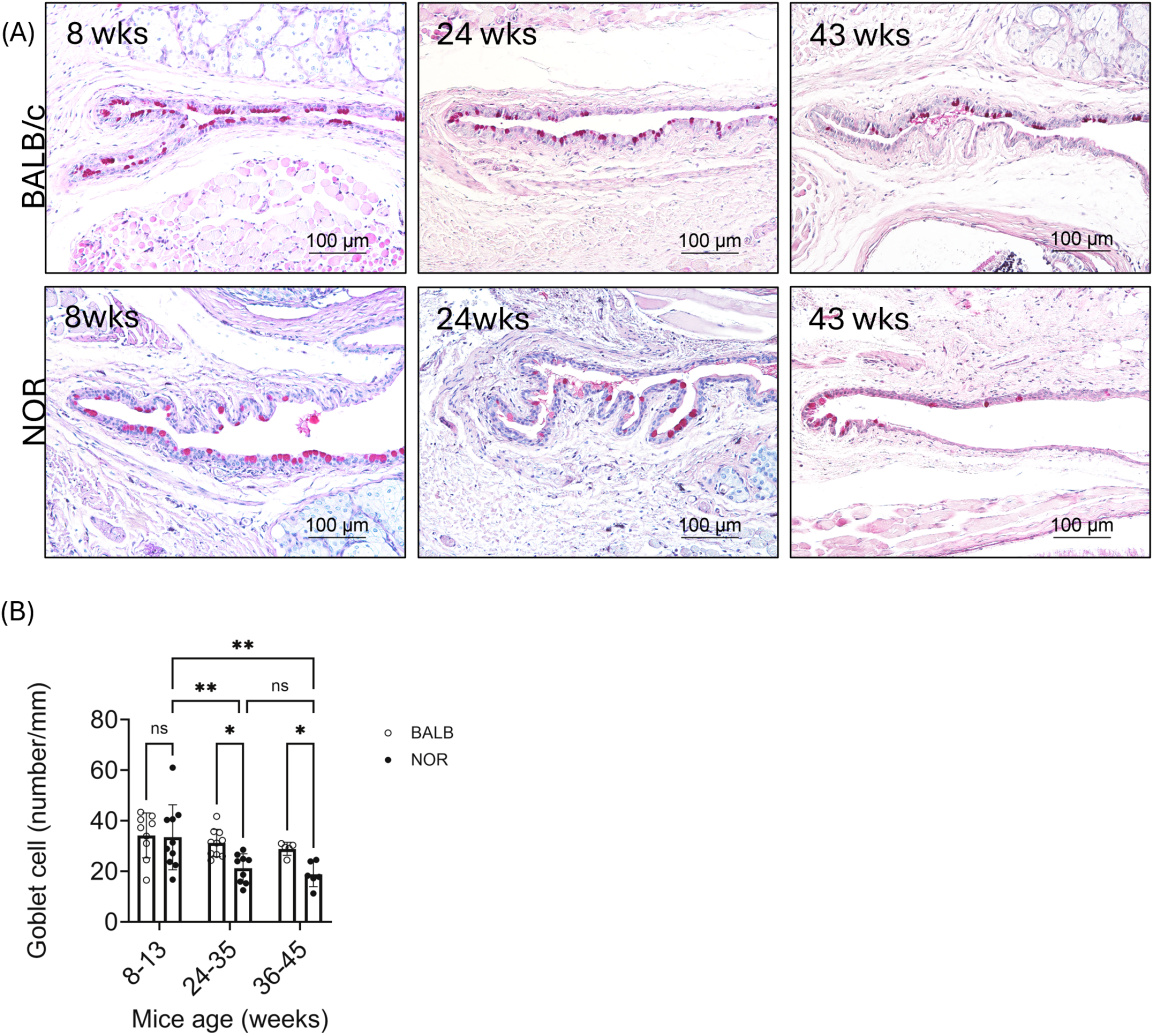
Decreased goblet cell density in NOR mice is indicative of SjD-associated ocular surface damage. **(A)** Representative images of conjunctival sections showing periodic acid-Schiff Stain (PAS) positive goblet cells (dark pink) in conjunctivas obtained from male NOR and BALB/c mice at different ages. **(B)** Bar graphs showing the mean ± SD number of PAS+ goblet cells/mm in male BALB/c and NOR mouse conjunctivas from mice at different ages, counted by light microscope and expressed per millimeter tissue. Each data point was generated from goblet cell counts from left and right eyes which were used as independent data points (the number of mice used per group is 3–5 mice). Results presented as mean ± SD. Statistical significance is indicated as *P<0.05, **P<0.01.

We previously showed that male NOR mice have reduced stimulated tear flow by 20 weeks of age (10), but further temporal information regarding the relationship between LG ELS formation and SjD-associated ocular surface disease was lacking in this model. Goblet cells produce mucins, responsible for lubrication of the ocular surface and prevention of infection (33), while inflammation of the conjunctiva associated with reduced goblet cell density is reported in SjD patients (34). The number of PAS-positive mucin-filled goblet cells in conjunctival samples from male NOR mice and BALB/c mice were thus quantified (**Figure 8**). At 8–13 weeks of age, conjunctival goblet cell density was not significantly different between the strains, but by 24–35 weeks of age, conjunctival goblet cell density was significantly decreased in NOR mice relative to both 24–35 week BALB/c mice (P-value<0.05) and 8–13 week NOR mice (P-value<0.006). A Sidaks multiple comparison test showed that conjunctival goblet cell loss occurred more slowly than did ELS formation in the LG, since no loss of goblet cells occurred by 8–13 weeks of age in NOR mice while ELS formation was already notable in the LG. To see if goblet cell density further decreased with age, we added older mice (36–45 weeks) to the analysis. Goblet cell density remained significantly decreased in male NOR mouse conjunctiva relative to male BALB/c mouse conjunctiva (P-value<0.05) but was not significantly reduced relative to the density measured in 24–35 week NOR mice. Thus, ocular surface disease, as reflected by reduced density of conjunctival goblet cells, lagged behind development of LG ELS but was fully manifested by 24–35 weeks. A trend to an age-related decrease in conjunctival goblet cell density was also noted in BALB/c mice, although it was not statistically significant.

## Discussion

Murine models are powerful tools for studying SjD, providing valuable insights into disease mechanisms associated with immunological dysregulation across different disease stages (35). NOD mice are extensively used to study SjD etiology and to test new therapies, due to their ability to reproduce key features of human SjD (36). However, NOD mice spontaneously develop diabetes. Because hyperglycemia-related inflammation can influence immune activation and exocrine gland secretion, this development may mask true SjD-related pathology. In support of this, Allushi and colleagues have reported that SG dysfunction in female NOD mice is strongly associated with hyperglycemia and the systemic elevation of pro-inflammatory cytokines (37). For this reason, we focused on NOR mice, a NOD-derived diabetes-free model that spontaneously develops SjD-like autoimmune exocrinopathy (10, 38).

To date, only a limited number of studies have been performed on NOR mice within the context of SjD (10, 39, 40). Other diabetes-free NOD-derived mice models prone to SjD have been developed through the generation of congenic strains (14, 41, 42). The double congenic C57BL/6.NOD-*Aec1Aec2* strain is a NOD derived strain in which the *Idd3* and *Idd5* regions of chromosome 3 and chromosome 1, respectively, were bred into C57BL/6 mice. This strain develops robust and rapid SjD-like lymphocytic infiltration, secretory dysfunction and autoantibodies (42). Analysis of disease in female mice of this strain showed lymphocytic infiltration of the LG by 20 weeks, with an earlier significant reduction in the number of goblet cells by 12 weeks (43). Another related strain, the C57BL/6.NOD-Aec1R1Aec2, demonstrated a full SjD-like phenotype including lymphocytic infiltration in both LG and SG of male mice accompanied by reduced tear and saliva flow, while female mice lacked LG disease but exhibited SG disease (44). Unlike these strains, NOR mice are a spontaneous disease model sharing approximately 88% of their genome with the NOD strain (including the *H2^g7^* MHC allele). Their use allowed analysis of the disease over an extended time course while capturing a large portion of the immune complexity inherent in the spontaneous NOD model, rather than studying features that arise from specific loci (45). In addition to the detection of structural components of HEV and FDC in LG ELS from male NOD mice (39), the distinct spectrum of autoantibodies present in tears from male NOD and male NOR mice are comparable (39). Although the pro-inflammatory environment associated with hyperglycemia may accelerate ELS formation in the NOD mouse, the nature of the ELS that ultimately form in the LG between NOD and NOR mice appear comparable.

ELS organization is categorized into three stages: (i) formation of immature lymphoid aggregates with minimal B/T cell organization in the absence of other defining morphological elements; (ii) formation of intermediate ELS with segregated B/T cells, the presence of some FDC and formation of small PNAd^+^ HEV; and (iii) formation of mature ELS with segregated B/T cells, a rich FDC network and extensive PNAd^+^ HEV (46, 47). ELS exhibiting these characteristics may be present at all disease stages, but differ in their abundance and dimensions. In the LG of male NOR mice, some mature ELS were seen by 8 weeks of age but they were smaller and less abundant than the mature ELS seen in the 24-week male NOR mouse LG. ELS across the three disease stages in the LG showed roughly equal percentages of B and T cells in the infiltrates. In contrast, studies in human SG have revealed that T cells in ELS initially outnumber B cell in small immune foci, followed by the formation of more severe lesions where the B to T cell ratio increases (48).

**Scheme 1:**
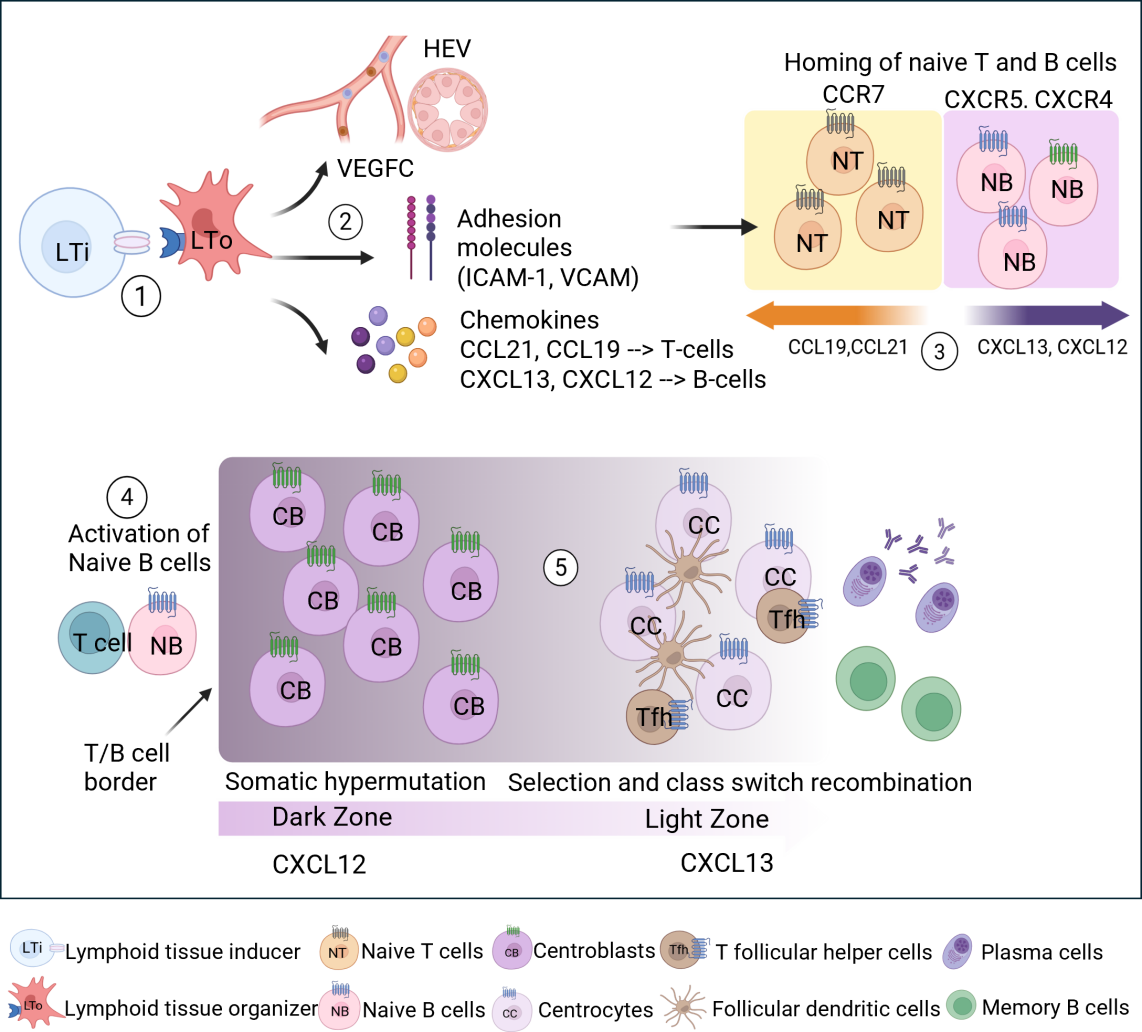
Illustration of typical SLO germinal center neogenesis highlighting commonalities with features of ELS in LG from the male NOR model of SjD. (1) Activation of the lymphotoxin signaling pathway via interaction between lymphoid tissue-inducing (LTi) cells with local lymphoid tissue organizer (LTo) occurs through binding of lymphotoxin α1β2 (LTα1β2) to LTβ receptor (LTβR). (2) Signaling pathways initiate expression of growth factors, adhesion molecules, and inflammatory chemokines, resulting in the formation of HEV and the subsequent homing of naïve B and T cells to the target site. (3) Localization of different chemokines allows segregation of B and T cells to their respective zones. (4) Activation of naïve B cells by antigen-experienced T cells at the T/B cell border region before entering the germinal center. (5) Differential abundance of CXCL12 and CXCL13 results in the presence of a dark zone composed of highly proliferative centroblasts expressing complementary CXCR4 receptor and a light zone composed of centrocytes undergoing selection and class switch recombination by AID enzyme and T follicular helper cells and lastly differentiate into memory B cells and antibody-producing plasma cells. In accordance with the findings in the male NOR model of autoimmune dacryoadenitis, early disease (8 weeks) was characterized by high levels of adhesion, homing and pro-inflammatory molecules of ICAM-1 and TNF, accompanied by increased gene expression of *Ccl19, Ccr7, Cxcr5*, and HEV-associated genes) priming tissue for lymphocytic recruitment and consistent with the early presence of apparent ELS. This was followed by increased expression of *Cxcl13, Fasl* and *Ctss* by 12 weeks indicating a later recruitment of B cells and concurrent activation of apoptosis along with increased extracellular remodeling and antigen presentation. By 16 weeks, increased *Il21* could promote germinal center like organization along with reduced *Gpx4* and *Aqp5* indicating increased epithelial oxidative stress, ferroptosis and reduced secretory functions. Increased *Acida* levels were observed in later disease by 20 weeks suggesting that by this stage somatic hypermutation and class switch recombination is occurring in germinal center-like structures. Increased IgG producing plasma cells are seen in the LG by 24 weeks, suggesting autoantibody production. Created by BioRender.com.

Our working model for ELS development in the LG (**Scheme 1**) is based on the sequence of events reported to occur in SLOs during germinal center neogenesis. The lymphotoxin axis, essential for lymphoid organogenesis, depends on interactions between VCAM-1^+^ICAM-1^+^LTβR^+^ mesenchymal lymphoid tissue organizer (LTo) cells and CD3^-^CD4^+^CD45^+^IL-7Ra^+^RANK^+^ lymphoid tissue inducer (LTi) cells (**Scheme 1**- Step 1). The latter express LTα1β2, a heterotrimeric cytotoxic factor composed of LTα and LTβ, which binds and activates lymphotoxin β receptor (LTβR) present in LTo (49). Blockage of LTβR can reduce lymphocytic infiltration due to reduced PNAd and MAdCAM present on high endothelial venules (HEV) (50). However, postnatal ELS may not rely on the conventional LTi driven pathway described for SLO, but rather use a lymphoid chemokine/LTβ pathway regulated by distinct inflammatory and stromal progenitors (immunofibroblasts) (51–55). Consistent with this, *Ltb gene* expression in male NOR mouse LG was elevated with disease progression, along with elevated expression of subsequent chemokines (*Cxcl3* and *Ccl19*). This finding suggests activation of the LTβR signaling pathway in LG ELS despite the lack of changes in *Ltbr* gene expression.

Activation of LTβR signaling upregulates chemokines such as CXCL13, CCL19 and CCL21 along with adhesion molecules and vascular endothelial growth factors that increase vasculature via formation of HEV (56) (**Scheme 1**- Step 2). HEV were detected in LG ELS, lined with the characteristic plumped cuboidal endothelial cells that express PNAd, a ligand for L-selectin on lymphocytes which facilitates trafficking of naïve lymphocytes from the blood stream to target organs (57). Diseased LG show early increased gene expression of *Cxcl13* and *Ccl19* and their receptors (*Cxcr5* and *Ccr7*) present on B and T cells, respectively, that facilitate compartmentalization of B and T cell zones (**Scheme 1**-Step 3) and enable interaction between naïve B cells and antigen-experienced T cells (**Scheme 1**- Step 4) (58). These findings are consistent with earlier work performed on SG in an induced mouse model of SjD (8).

In SLO, activated B cells expressing CXCR4 migrate toward the DZ rich in CXCL12 where they are referred to centroblasts and undergo hyperproliferation and somatic hypermutation by activation-induced cytidine deaminase (AID) enzyme; this event is followed by cellular migration to the CXCL13 abundant LZ via CXCR5 upregulation (59). In the LZ, these cells undergo selection and affinity maturation by FDC and T follicular helper cells and terminal differentiation into antibody producing plasma cells or memory B cells (**Scheme 1**- Step 5) (1, 21). Histological evidence of the presence of DZ and LZ is seen in LG ELS across all disease stages (**Figure 1**, **Supplementary Figure 2**). ELS in the LG exhibited additional characteristic features associated with functional germinal centers indicating the presence of functional B cell activation (increased gene expression of *Aicda)*, the presence of an FDC cell network within B cell follicles, and the presence of GL7+ germinal center B cells (8, 55). This identification of germinal center like functions in LG ELS suggests the potential for local immune hyperactivity and production of autoantibodies potentially linked to risk of B-cell lymphoma (55).

Plasma cells expressing IgA are abundant in healthy exocrine glands, producing antibodies transported to tears and saliva to bolster mucosal immunity. Elevated levels of IgG-producing plasma cells are reported in serum and SG biopsies from SjD patients with advanced disease (20, 60). Using published data from a scRNA-Seq study of LG from the NOD.H2b disease model, we have previously confirmed high local IgG production in the LG (39, 61). In the current study, we detected a greater number of plasma cells containing IgG in the LG of NOR mice with established disease (24 weeks) relative to early disease (8 weeks), suggesting that local IgG production was increased as ELS occupancy of the LG increased. Our previous analysis of different IgG autoantibodies elevated in tears versus serum from male NOR mice utilizing an autoantibody microarray suggested that autoantibody profiles were distinct in these biofluids (39). Elevated tear IgG autoantibodies in male NOR mice (Jo1, IA2, tTG, Mi-2, TPO and SAE1/SAE2) were likely produced from LG ELS. These autoantibodies, unlike SSA and SSB, require special techniques for measurement that we intend to explore in future studies. However, increased LG ELS occupancy of the LG in male NOR mice was correlated with increased total IgG levels in LG lysates (**Figures 5G–I**, **Supplementary Figure 3A**).

Although apoptosis is a part of cellular homeostasis and normal tissue turnover, it can also induce autoimmune responses via impairment of efficient phagocytosis of apoptotic cells. This can result in accumulation or release of autoantigens in apoptotic blebs, leading to production of autoantibodies (62). This possibility is supported by the significant upregulation in gene expression of *Fasl* detected in the LG by 12 weeks of age. Previous studies have shown that SG acinar epithelial cells from SjD patients show high expression of Fas and FasL, leading to increased cell death (63). Another newly identified form of cell death, triggered by dysregulated lipid peroxidation and accumulation of reactive oxygen species (ROS) is ferroptosis (24). Previous transcriptome analysis studies on SjD human and mouse SG showed differential expression of ferroptosis-related markers, including upregulation of IFN-γ and downregulation of glutathione peroxidase 4 (GPX4) and aquaporin 5 (64). GPX4 is a key regulator of ferroptosis, which utilizes glutathione to detoxify hydroperoxides (65). It is reported that lipid ROS accumulation resulting from GPX4 downregulation leads to increased phosphorylation of STAT4, its subsequent nuclear translocation, and binding to the aquaporin-5 promotor inhibiting aquaporin 5 gene expression (25). Increased ferroptosis in the LG of diseased NOR mice are supported through decreased gene expression of both *Gpx4* and *Aqp5* by 16 weeks.

Formation of ELS in the LG would be expected to be associated with changes in tear flow and composition, leading to loss of ocular surface integrity. Reduction in conjunctival goblet cell numbers is associated with dry eye diseases including SjD-associated dry eye (33, 66). Relative to the early time course of ELS formation in the LG of male NOR mice which was notable by 8 weeks, a significant reduction in conjunctival goblet cell density was not seen in mice until 24–35 weeks. Conjunctival goblet cell loss in SjD has been attributed to persistence and accumulation of IFN-γ and chemokines induced by IFN-γ (i.e. CXCL9, CXCL10, CXCL11) in addition to increased tear hyperosmolarity, causing cell death of mucin-producing goblet cells (67, 68).

While SjD is more prevalent in female patients, male patients may have more severe ocular complications and systemic manifestations (69). The protective effects of ovarian hormones on SjD in the LG were confirmed in a study conducted by Czerwinski et al., showing that ovariectomized NOD.B10.H2^b^ mice exhibited accelerated disease progression (70). The NOD.B10.H2^b^ strain, like the NOR, is a NOD-derived mouse strain in which the unique I-Ag7 segment of the MHC molecule of the NOD strain essential for development of insulitis and diabetes was replaced with I-Ab from a non-diabetic B10 strain (41, 71).

Comprehensive characterization of LG ELS in SjD is limited, in contrast to the extensive work conducted in SG in murine models of SjD (8, 55, 72, 73). The LG and SG differ in their unique structure, development and function (6, 74). A recent study evaluating the presence of ELS in the LG of aged mice, a model of age-related DED showed similarity to SjD pathogenesis in pathways related to adaptive immune responses, leukocyte activation and cytokine production (75). Studies comparing ELS development in LG and SG within the same experimental model are scarce. Truman et al. have investigated the effects of localized expression of lymphotoxins on formation of tertiary lymphoid structures in both LG and SG using a transgenic mouse model (Amy1-LTαβ). This study showed that lymphotoxins promoted development of well-organized ELS in both glands but did not compare differences in structural organization and time course of formation of ELS between the glands (76). To begin to understand these possible differences, we used an identical approach to evaluate ELS formation and progression by IF and to measure changes in genes associated with ELS formation in SMG of the female NOR mouse. Analysis of SMG infiltration in mice aged 8–32 weeks revealed that only a few defined B/T cell aggregates were detectable in the SMG at 8 and 16 weeks of age (**Supplementary Figure 5A**). These B/T cell aggregates in the female NOR mouse SMG increased in size and number over time, but remained relatively confined, smaller and exhibited less apparent fusion with adjacent ELS even by 32 weeks of age. HEV and FDC were detected in these aggregates. Analysis of changes in the gene expression of a subset of ELS related genes that were all significantly elevated between 8–12 weeks in the LG of male NOR mice showed that the earliest significant upregulation in this panel was observed for *Ltb* by 16 weeks of age (**Supplementary Figure 5B**), followed by *Glycam1* at 24 weeks (**Supplementary Figure 5C**) and *Cxcr5*, *Ccl19* and *Ccr7* only by 32 weeks of age (**Supplementary Figures 5D–F**). *Cxcl13* was not significantly elevated in female NOR mouse SMG at any time point evaluated (**Supplementary Figure 5G**). These findings suggest that the sexually dimorphic autoimmune exocrinopathy in the NOR strain is characterized by more rapid and extensive ELS formation in male mouse LG and slower and less extensive ELS formation in female mouse SMG. Given the observed protective effects of ovarian hormones on SjD-like LG pathology observed previously (70), further mechanistic and clinical studies exploring sex effects on exocrine pathology are warranted.

Definitive identification of ELS in human LG via histological analysis is currently challenging, due to limitations in the ability to biopsy the LG. New non-invasive imaging methods may ultimately be capable of quantifying LG ELS in patients to better evaluate their correlation with clinical symptoms, but this capacity is not currently in place. A few studies have specifically evaluated LG ELS and ocular symptoms of SjD in murine models. In the Amy1-LTαβ murine SjD model, the presence of ELS in LG was associated with glandular atrophy and significantly reduced tear production (76). Blockade of the LTBR pathway in NOD mice disrupted ELS-like structures in parallel with increased tear production and preservation of ocular surface integrity (77). Altogether, these findings suggest that LG ELS are important in generating ocular clinical manifestations. Further study of the complex interactions governing formation and sustainment of LG ELS may ultimately aid in design of new therapies which target ELS to more effectively treat LG gland pathology and SjD-associated dry eye disease.

## Data Availability

The original contributions presented in the study are included in the article/**Supplementary Material**. Further inquiries can be directed to the corresponding author.
