# Supplementary material for: Dynamic progression of ectopic lymphoid structure formation in lacrimal glands of a Sjögren’s disease murine model
Source: Front Immunol. 2026 Mar 19;17:1797691. doi: 10.3389/fimmu.2026.1797691 (PMC13044039; doi:10.3389/fimmu.2026.1797691)
Supplement: Supplementary file 1 [file Table1.docx]

Supplemental Material

# Supplemental Figures:

## Supplemental Figures


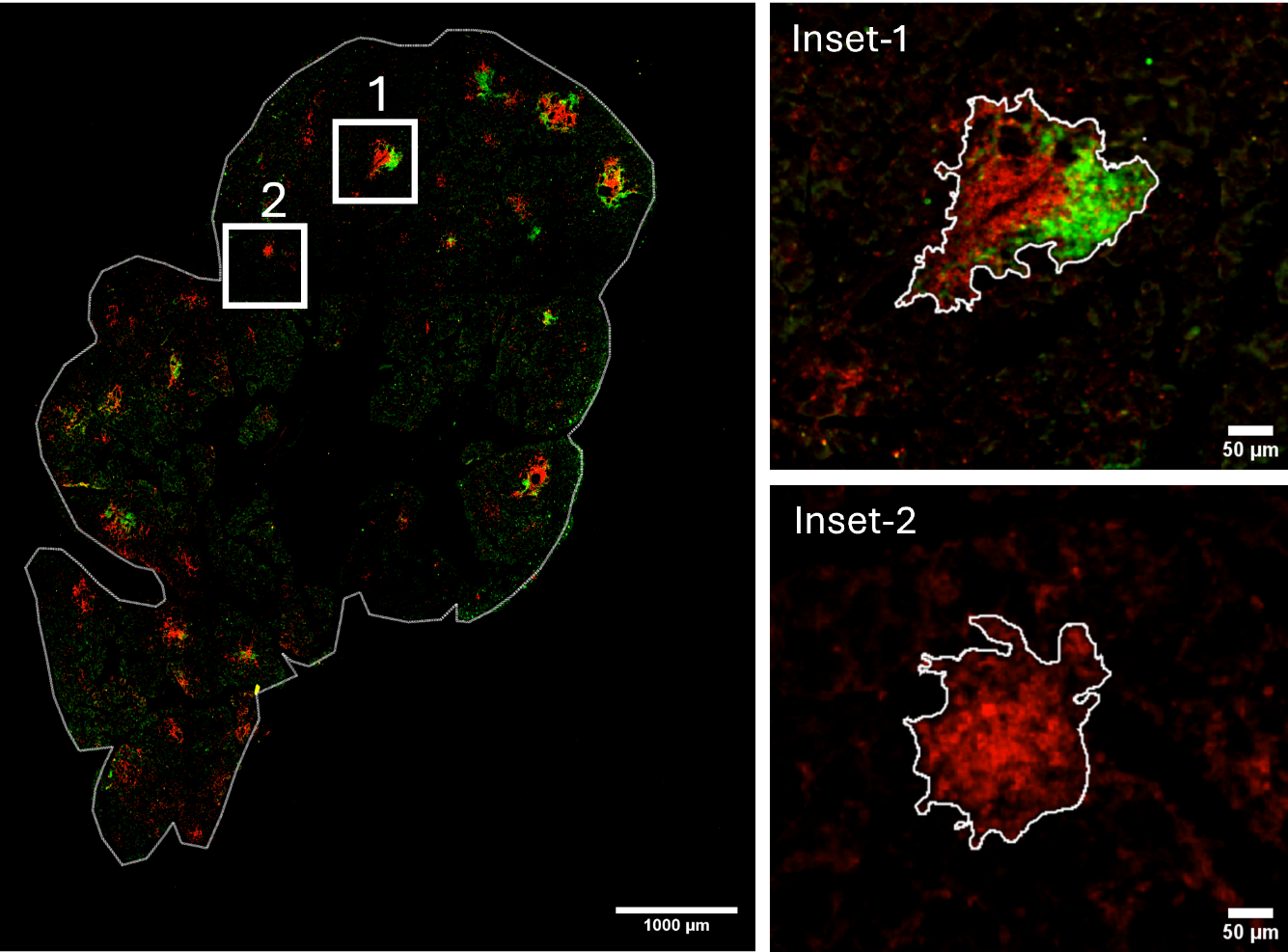


**Supplemental Figure 1**: Representative IF image showing B/T cell aggregates in a LG section from a 16-week male NOR mouse using antibodies to IgD to label naive B cells (green) and to CD3 to label T cells (red). Scale bar, 1000 μm. Inset-1 shows B/T cells compartmentalization in a typical aggregate while inset 2 shows an accumulation of T cells in an area lacking B cell accumulation. Inset scale bars, 50 μm. Areas in insets 1 and 2 were outlined using the “Magic Wand” tool using QuPath software (version 0.5.1.).


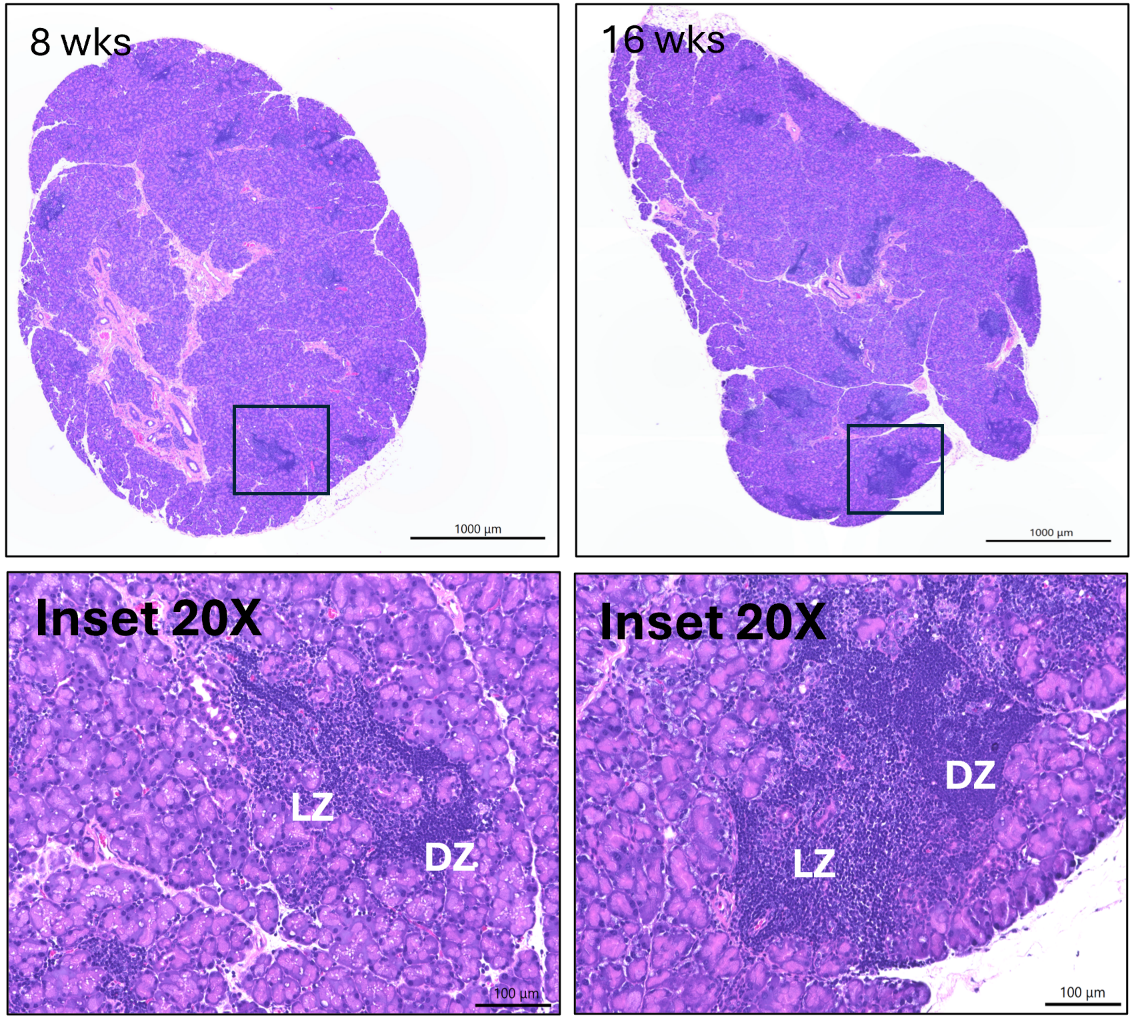


**Supplemental Figure 2:** Representative images showing lymphocytic infiltration in LG from male NOR mice aged 8 wks and 16 wks by H&E staining of LG sections. Scale bar, 1000 μm. The boxed regions in the images are shown in the Inset panels below. Insets show 20X magnification (Scale bar, 100 μm) of regions showing compartmentalization of foci into dark zones (DZ) and light zones (LZ). N=3 LG from 3 mice per age group.


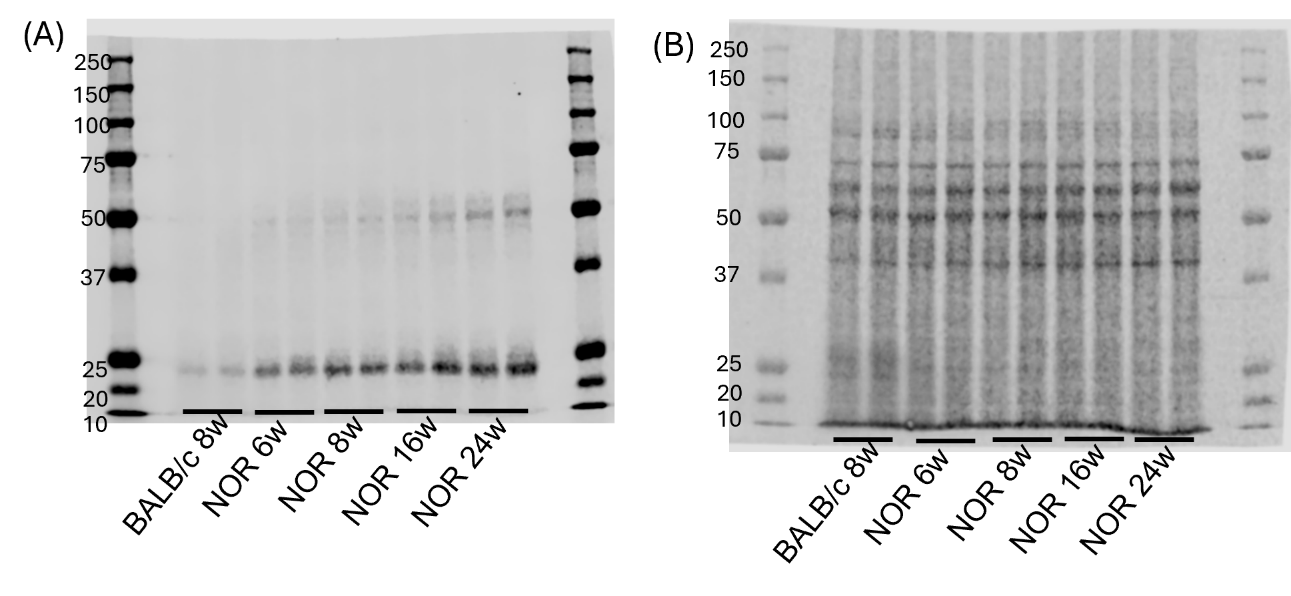


**Supplemental Figure 3:** Representative Western blots showing light chain (LC) and heavy chain (HC) IgG signal in LG lysates from male BALB/c and male NOR mice in parallel with total protein stain in the same samples. (A) Western blot showing increasing intensity of bands at 25KDa and 50KDa corresponding to LC and HC, respectively, obtained by blotting with a secondary donkey anti-mouse antibody conjugated to IRdye 680 as described in Methods. (B) Nitrocellulose membrane showing total protein stain obtained with Revert 700 total protein stain and used for normalization of Western blot signal. 40 ug protein from LG lysates was loaded per lane. Lanes correspond to LG lysates from: male BALB/c 8 weeks (lane 1,2); male NOR 6 weeks (lane 3,4); male NOR 8 weeks (lane 5,6); male NOR 16 weeks (lane 7,8); and male NOR 24 weeks (lane 9,10).


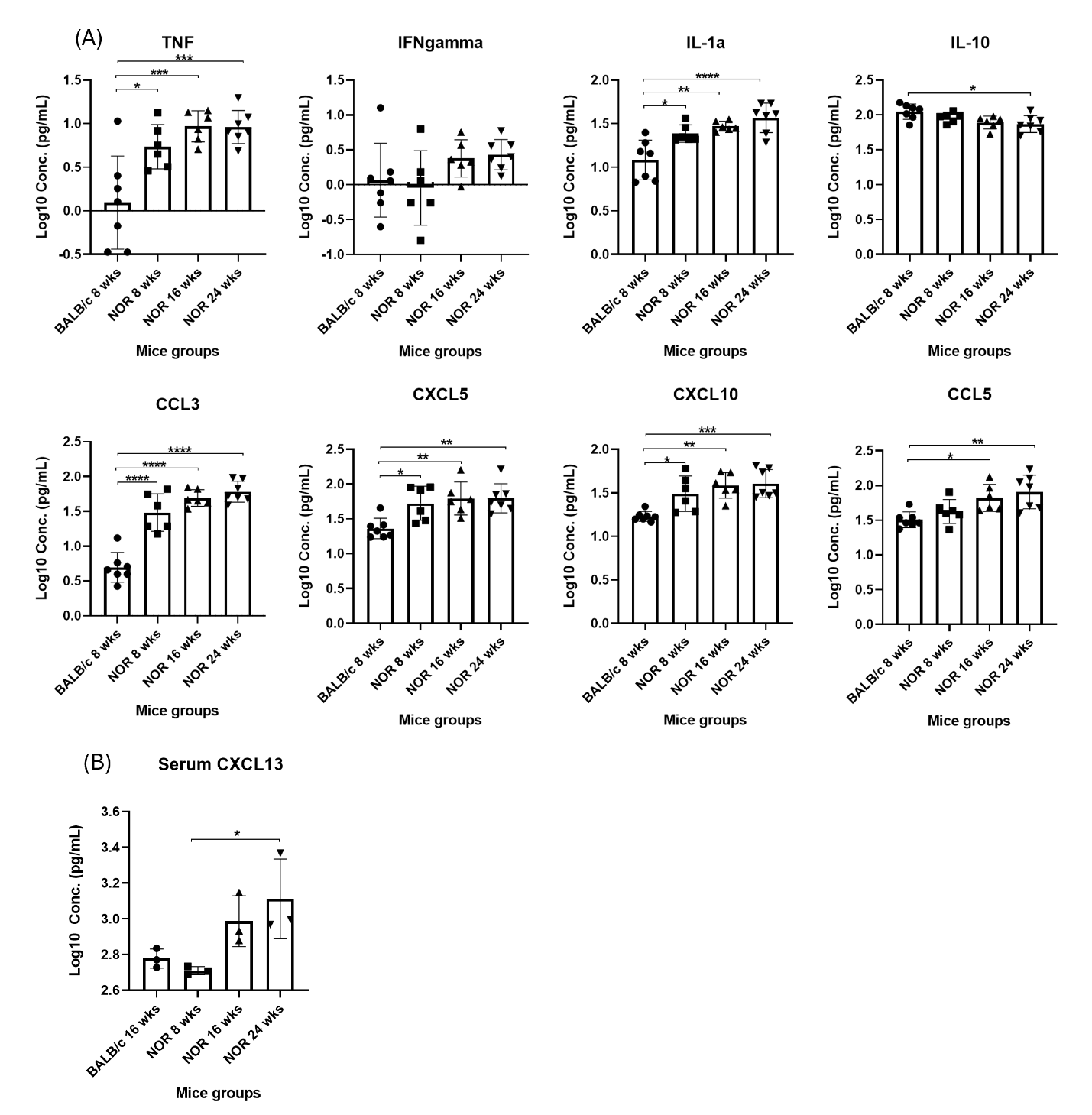


**Supplemental Figure 4**: ELISA measurements of local and systemic concentrations of pro-inflammatory cytokines and chemokines involved in inflammation and recruitment/homing of immune cells. (A) Cytokines and chemokines were assayed in LG tissue homogenates from male BALB/c and NOR mice at the indicated ages using a multiplex Luminex platform. (N=6-7 LG from 6-7 mice per group). (B) Serum concentration of CXCL13 in male BALB/c and NOR mice at the indicated ages. N=3 LG from 3 mice per group. Results presented are mean ± SD Statistical significance is indicated as *P<0.05, **P<0.01, ***P<0.001, ****P<0.0001.


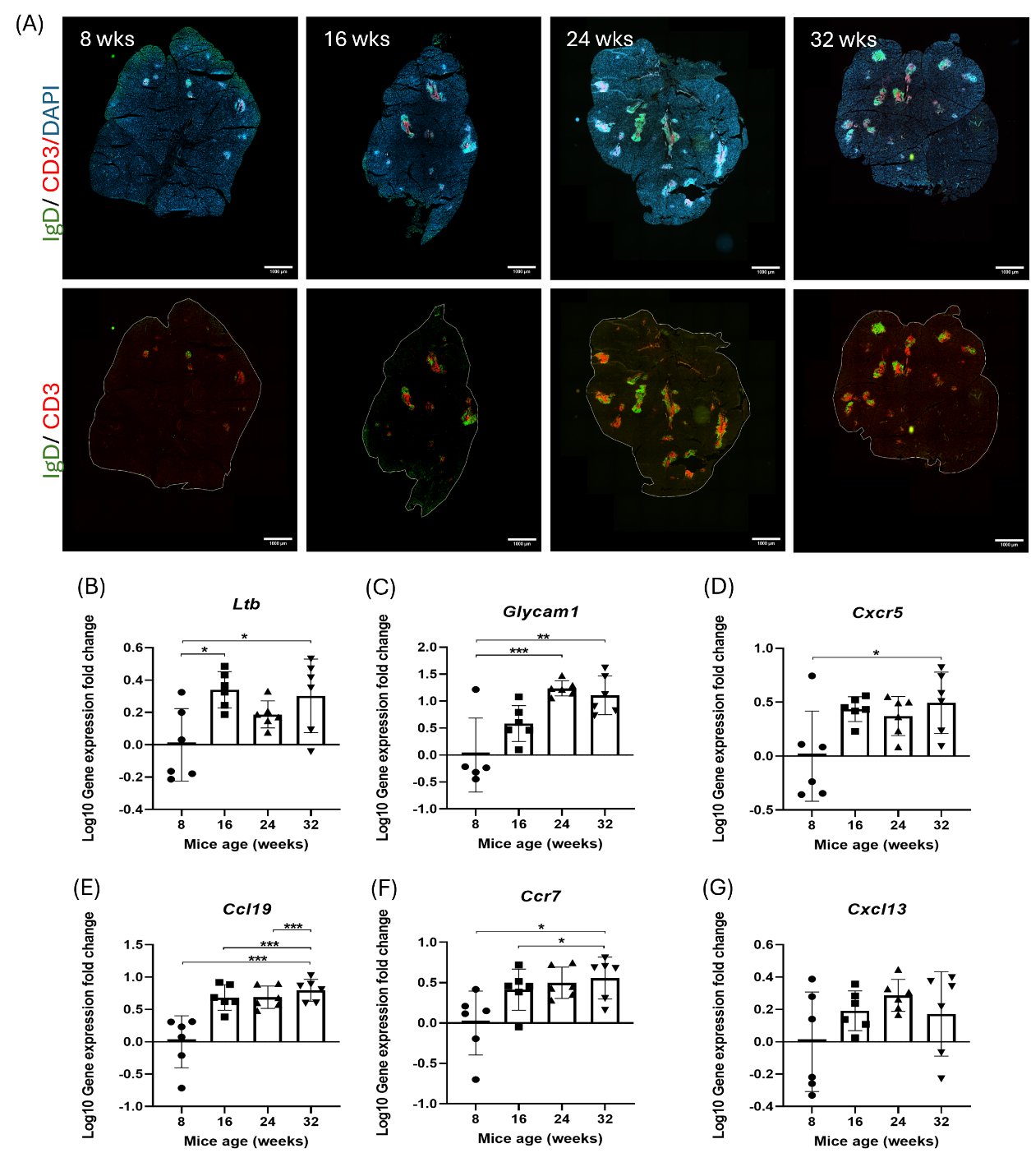


**Supplemental Figure 5:** Characterization of ELS development in SMG of female mice at early (8 and 12 weeks), intermediate (20 weeks), and established (32 weeks) disease. (A) Representative IF images show components of B/T cell aggregates using antibodies to IgD, highlighting naive B cells (green) and CD3, highlighting T cells (red). DAPI labels nuclei (blue). Scale bar, 1000 μm. Results are represented from N=6 SMG from 6 mice per age group. (B-G). Relative expression of ELS-related genes in SMG of female NOR mice. Genes include (B) *Ltb*, involved in the formation of lymphoid tissue; (C) *Glycam1*, High endothelial venule marker; (D) *Cxcr5*, chemokine receptor for CXCL13; (E) *Ccl19*, chemoattractant for naïve T cells; (F) *Ccr7*, chemokine receptor for CCL19; and (G) *Cxcl13*, chemotactic for subsets of B and T cells. N=6 SMG from 6 mice per age group, with each data point representing one SMG from one mouse. Results presented as mean ± SD. Statistical significance is indicated as *P<0.05, **P<0.01, ***P<0.001.

**Supplemental Tables:**

| **Primary Antibodies** | | | | | | |
| --- | --- | --- | --- | --- | --- | --- |
| **Antigen** | **Host** | **Dilution** | **Clone** | **Catalogue #** | **Source** | **Marker** |
| CD3e | Hamster | 1:200 | 500A2 | 553238 | BD Biosciences | T-cell |
| IgD | Rat | 1:200 | 11-26c.2a | 405708 | Biolegend | Naïve B cell |
| B220 | Rabbit | 1:200 | RA3-6B2 | MA5-48137 | Thermofisher Scientific | B cell marker |
| CD21/CD35 | Rat | 1:200 | eBio8D9 | 13-0211-82 | Thermofisher Scientific | FDC |
| PNAd | Rat | 1:200 | MECA-79 | 553863 | BD Biosciences | Glycam-1 in HEV |
| GL7 | Rat | 1:100 | GL7 | 14-5902-82 | Thermofisher Scientific | functional germinal center B cell |
| CD138 | Goat | 1:50 | - | AF3190 | R&D systems | Plasma cells |
| ICAM-1 | Goat | 1:100 | - | AF796 | R&D systems | ICAM-1 |
| **Secondary Antibodies** | | | | | | |
| **Host** | **Raised in** | **Dilution** |  | **Catalogue #** | **Source** | **Conjugation** |
| Goat | Hamster | 1:200 | - | A-21112 | Thermofisher Scientific | Alexa Fluor® 568 |
| Donkey | Rat | 1:200 | - | A-21208 | Thermofisher Scientific | Alexa Fluor® Plus 488 |
| Donkey | Goat | 1:200 | - | A-11055 | Thermofisher Scientific | Alexa Fluor® 488 |
| Goat | Rabbit | 1:200 | - | A-11034 | Thermofisher Scientific | Alexa Fluor® 488 |
| Mouse | Donkey | 1:2000 | - | 926-68072 | LICORbio | IRDye 680RD |
| **Additional Probes** | | | | | | |
| **Probe** | **-** | **Dilution** |  | **Catalogue #** | **Source** | **Conjugation** |
| Phalloidin | **-** | 1:200 | - | A30107 | Thermofisher Scientific | Alexa Fluor™ Plus 647 |
| DAPI | **-** | 1:1000 | - | D1306 | Thermofisher Scientific | **-** |

**Supplemental Table 1**: Primary and Secondary antibodies and additional probes used in immunofluorescent staining.

**Supplemental Table 2**: Genes and specific Taqman® primers and probes used in RT-qPCR.

| **Gene Product** | **Gene Expression Assay Identification** | **Source** |
| --- | --- | --- |
| *Glycam1* | Mm00801716_m1 | Thermofisher Scientific |
| *Cxcl13* | Mm00444533_m1 | Thermofisher Scientific |
| *Cxcr5* | Mm00432086_m1 | Thermofisher Scientific |
| *Ltb* | Mm00434774_g1 | Thermofisher Scientific |
| *Ltbr* | Mm00440235_m1 | Thermofisher Scientific |
| *Aicda* | Mm00507774_m1 | Thermofisher Scientific |
| *Ccr7* | Mm00432608_m1 | Thermofisher Scientific |
| *Ccl19* | Mm00839967_g1 | Thermofisher Scientific |
| *Il21* | Mm00517640_m1 | Thermofisher Scientific |
| *Aqp5* | Mm00437578_m1 | Thermofisher Scientific |
| *Gpx4* | Mm04411498_m1 | Thermofisher Scientific |
| *Ctss* | Mm01255859 | Thermofisher Scientific |
| *Fasl* | Mm00438864_m1 | Thermofisher Scientific |
| *Icam1* | Mm00516023 | Thermofisher Scientific |
| *Gapdh* | Mm99999915_g1 | Thermofisher Scientific |
